# An integrative, multi-omics approach towards the prioritization of *Klebsiella pneumoniae* drug targets

**DOI:** 10.1038/s41598-018-28916-7

**Published:** 2018-07-17

**Authors:** Pablo Ivan Pereira Ramos, Darío Fernández Do Porto, Esteban Lanzarotti, Ezequiel J. Sosa, Germán Burguener, Agustín M. Pardo, Cecilia C. Klein, Marie-France Sagot, Ana Tereza R. de Vasconcelos, Ana Cristina Gales, Marcelo Marti, Adrián G. Turjanski, Marisa F. Nicolás

**Affiliations:** 1 Instituto Gonçalo Moniz, Fundação Oswaldo Cruz (FIOCRUZ), Salvador, Bahia Brazil; 2 Laboratório Nacional de Computação Científica, Petrópolis, Rio de Janeiro, Brazil; 3 Plataforma de Bioinformática Argentina (BIA), Instituto de Cálculo, Facultad de Ciencias Exactas y Naturales, Universidad de Buenos Aires, Buenos Aires, Argentina; 4 Departamento de Química Biológica, Facultad de Ciencias Exactas y Naturales, Universidad de Buenos Aires, Ciudad Universitaria, Pabellón 2, C1428EHA Ciudad de Buenos Aires, Argentina; 5 Instituto de Química Biológica de la Facultad de Ciencias Exactas y Naturales (IQUIBICEN) CONICET, Ciudad Universitaria, Pabellón 2, C1428EHA Ciudad de Buenos Aires, Argentina; 6 Inria Grenoble Rhône-Alpes, Grenoble, France; 7 Université Claude Bernard Lyon 1, Lyon, France; 8 Laboratório Alerta. Division of Infectious Diseases, Department of Internal Medicine. Escola Paulista de Medicina, Universidade Federal de São Paulo, São Paulo, Brazil; 9 Present Address: Centre for Genomic Regulation (CRG), Departament de Genètica, Microbiologia i Estadística, Facultat de Biologia and Institut de Biomedicina (IBUB), Universitat de Barcelona, Barcelona, Catalonia, Spain

## Abstract

*Klebsiella pneumoniae* (*Kp*) is a globally disseminated opportunistic pathogen that can cause life-threatening infections. It has been found as the culprit of many infection outbreaks in hospital environments, being particularly aggressive towards newborns and adults under intensive care. Many *Kp* strains produce extended-spectrum β-lactamases, enzymes that promote resistance against antibiotics used to fight these infections. The presence of other resistance determinants leading to multidrug-resistance also limit therapeutic options, and the use of ‘last-resort’ drugs, such as polymyxins, is not uncommon. The global emergence and spread of resistant strains underline the need for novel antimicrobials against *Kp* and related bacterial pathogens. To tackle this great challenge, we generated multiple layers of ‘omics’ data related to *Kp* and prioritized proteins that could serve as attractive targets for antimicrobial development. Genomics, transcriptomics, structuromic and metabolic information were integrated in order to prioritize candidate targets, and this data compendium is freely available as a web server. Twenty-nine proteins with desirable characteristics from a drug development perspective were shortlisted, which participate in important processes such as lipid synthesis, cofactor production, and core metabolism. Collectively, our results point towards novel targets for the control of *Kp* and related bacterial pathogens.

## Introduction

Antibiotic resistance in bacteria represents a global health concern. Every year, over 136,000 deaths are attributable to infections caused by these type of microorganisms in healthcare settings in the USA and Europe alone^1^. Drug resistance can be associated to a multitude of factors that comprise the misuse of antimicrobials; poor-quality medicines; and insufficient regulation on the prescription of drugs^1^, issues that are easy to identify but complex to resolve. As an installed phenomenon, besides the focus on improving regulation policies, efforts to tackle antibiotic-resistant pathogens should also turn to the discovery of new compounds. However, we currently face a low output antibiotic development pipeline that, coupled to the unattractive costs and the regulatory challenges of developing and launching new drugs, led to many pharmaceutical companies exiting the field^2^. A major limitation of traditional high-throughput screening (HTS) approaches is that only a finite amount of chemicals in a limited number of conformations are available in any given HTS library. As this chemical space limitation will hardly be overcome, novel approaches are needed to tackle the rising problem of bacterial resistance to current treatments. Our study provides a framework for which such novel strategies can be developed and further adapted to use. With sheer amounts of high-throughput genomic, structural and transcriptomic data from many important bacterial pathogens openly available in biological databases, an approach based on multidimensional data integration towards pinpointing new drug targets represents a more rapid and cost-effective strategy than traditional screening techniques. In line with this, previous efforts have been employed to *in silico* detect drug targets in the proteomes of clinically relevant bacteria such as *Corynebacterium* spp.^3^, *Mycobacterium tuberculosis*^4–8^, *Streptococcus pneumoniae*^9^, *M. leprae*^10^, *Helicobacter pylori*^11^*, Clostridium botulinum*^12,13^, *Pseudomonas aeruginosa*^14^, *E. coli*^15^ and other *Enterobacteriaceae* family members^16^, as well as *Staphylococcus epidermidis*^17^. Building upon some of these genome mining works, others have successfully set out to find inhibitors of targets of interest, such as those acting on *S. aureus* wall teichoic acid biogenesis components (for which the identified compounds potentiated the action of β-lactams)^18^, quorum-sensing components in *P. aeruginosa*^19^, and histidine kinases of *Shigella flexneri*^20^ and *S. epidermidis*^21^. While the application of computational techniques alone does not envisage a definitive identification of drug targets, it does permit shortlisting more plausible targets, effectively reducing the search space to candidates with increased probability of serving as targets for either a new or a repositioned drug.

In this work, we concentrated our efforts towards target detection in the *Klebsiella pneumoniae* proteome. This non-motile, rod-shaped, Gram-negative enterobacterium occupies diverse ecological niches ranging from soil to water, but from a human health perspective, it represents one of the most important pathogenic bacteria^22,23^. *K. pneumoniae* is commonly reported as an etiologic agent of either community-acquired urinary tract infections or bacterial pneumonia. However, it can cause any type of infection in hospital settings, including outbreaks in newborns and adults under intensive care, which is likely associated with its ability to spread rapidly in the hospital milieu.

Among the *K. pneumoniae* antimicrobial resistance repertoire, the production of carbapenemase is particularly worrisome since it confers resistance to all beta-lactams. Infections caused by carbapenem-resistant *K. pneumoniae* represent a high burden of disease worldwide especially in countries like Argentina, Brazil, Colombia, Greece, Israel, and Italy, where KPC-2-producing *K. pneumoniae* are endemic. For example, according to the last report of the Brazilian Health Surveillance Agency, *Klebsiella* spp. was the most frequent microorganism causing 3,805 (16.9%) catheter-related bloodstream infections in adult patients hospitalized at the Brazilian intensive care units in 2015^24^. Nearly 43% of these isolates were resistant to both broad-spectrum cephalosporins and carbapenems^24^. In this way, polymyxins have become last resort antimicrobials for treatment of serious infections caused by KPC-2-producing *K. pneumoniae*. Unfortunately, to make things even worse, an important increase in the resistance rates to polymyxins has been observed among carbapenem-resistant *K. pneumoniae*. Braun and collaborators have observed an increase in polymyxin B resistance rates from 0 to 30.6% among  *K. pneumoniae* isolates recovered from blood cultures between 2009 and 2015 in a tertiary Brazilian hospital^25^. Similar results have been reported by Bartolleti *et al*.^26^. Because of this, the search for new strategies to counter these infections is ongoing, and include the use of novel approaches such as nanoparticles in combination with antibiotics^27^, as well as immunotherapy based on monoclonal antibodies targeted towards components of the bacterial outer polysaccharide capsule^28^.

In here, we report the application of a multidimensional data integration strategy in order to prioritize drug targets in *K. pneumoniae*. By combining various layers of information into a multi-omics approach, which included genomic, transcriptomic, metabolic and protein structural data sources, we were able to delineate candidate proteins with features that are relevant to target selection in *K. pneumoniae* and related pathogens. Particularly, we incorporated information about polymyxin resistance in our analyses, in order to enrich for targets that would also be useful against the so-called ‘superbugs’.

## Methods

### Bacterial strain and annotations

*Klebsiella pneumoniae* Kp13 (referred to as Kp13 throughout the text) was first isolated by our group during a nosocomial outbreak in an intensive care unit that occurred in 2009 in South Brazil. This strain is resistant to many antibiotics, including polymyxin B. We have previously reported minimum inhibitory concentrations (MICs) for Kp13 against several antibiotics^29^. In addition, Kp13 is a carbapenemase producer, harbouring the gene coding for KPC-2 in plasmid pKp13d^30^. Our group has determined its complete genome, which comprises one 5.3 Mbp circular chromosome and six plasmids (totalling 0.43 Mbp), and we have manually annotated its predicted coding sequences (CDS), composed of 5,736 predicted peptides^30^. All annotations and sequences for this bacterium are available at the BioProject/NCBI (https://www.ncbi.nlm.nih.gov/bioproject/) under accession no. PRJNA78291. Subcellular localization was predicted for each protein using PSORTb v3.0.2 running in Bacterial, Gram-negative mode^31^.

### Generation of structural homology-based models

394 unique crystal structures for *K. pneumoniae* proteins were retrieved from the Protein Data Bank (PDB)^32^. For all remaining predicted peptide sequences, we tried to build homology-based models using our previously developed structural genomic pipeline^33^. Protein sequences were used as input for PSI-BLAST searches (parameters -j 3 -e 1e-05)^34^ against the UniRef50 (UniProt trimmed at 50% redundancy)^35^. Once a position-specific scoring matrix (PSSM) was obtained, this PSSM was used to search against the PDB95 (non-redundant PDB at 95% level) using PSI-BLAST (parameter -e 1e-05). Up to five recovered PDB models were used as templates for the homology-based reconstruction, and MODELLER^36^ was employed to construct five models per template for each Kp13 protein. One representative model was chosen based on the GA341 score (>0.7) and maximization of the QMEAN Z-score function^37^. With this strategy, we were able to build a total of 3,194 structural models for *K. pneumoniae* Kp13 sequences.

### Classification of *K. pneumoniae* Kp13 proteins according to their druggability

The druggability concept describes the capacity of a peptide to bind to a drug, leading to protein modulation in a desired manner, such as inhibition in the case of antibiotic drugs. Part of our group has developed a methodology for druggability prediction^33^ based on the open source pocket detection algorithm *fpocket*^38^, which combines several physicochemical descriptors to estimate the pocket druggability and can be used on a large, genomic scale^3^. Based on previous analysis of druggability score (DS) distribution for all pockets that are found to host a drug-like compound in the PDB, pockets were classified into four categories: non-druggable (0.0 ≤ DS < 0.2), poorly druggable (0.2 ≤ DS < 0.5), druggable (0.5 ≤ DS < 0.7) and highly druggable (0.7 ≤ DS ≤ 1.0). All proteins for which we obtained structural models were subject to this classification (refer to http://target.sbg.qb.fiocen.uba.ar/patho/user/methodology for further details).

### Construction of the whole-genome metabolic network of *K. pneumoniae* Kp13

To build the metabolic network of Kp13 (referred as Kp13-MN), we used the PathoLogic module of Pathway Tools v. 18.0^39^. This tool accepts an annotated genome in Genbank format as input and creates a Pathway/Genome Database (PGDB) containing the predicted metabolic pathways of a given organism. Metabolic reconstruction included determining gene-protein-reaction associations, which are primarily based on the corresponding enzyme commission (EC) number. Annotation of metabolic enzymes was performed manually during the genomic annotation of Kp13 strain, described elsewhere^30^, and this annotation was complemented using PRIAM^40^. After automatic reconstruction, a detailed manual curation of the metabolic network was performed, which comprised the following steps: 1) inclusion of missing pathways with biological evidence for their presence; 2) removal of false positively predicted pathways; and 3) filling of enzymatic ‘holes’ in predicted but incomplete pathways assisted by the Pathway Hole Filler module within Pathway Tools. After the construction and curation processes, the metabolic network was exported in systems biology markup language (SBML) format for downstream analyses. Reactions involving macromolecules (such as DNA, RNA, and proteins, as per the BioCyc ontology) were filtered, as were transporter proteins, and only reactions involved in small-molecules metabolism were considered. The rationale for this strategy was that since most of the current antibiotics target macromolecules (such as proteins, ribosomes, lipids), the focus on enzymatic activities not related to these molecules would comprehend a largely unexplored universe suitable for the discovery of novel targets. With these premises, only 110 unique genes were left out of the analyses, being therefore of little impact considering the total metabolic and genomic space that was effectively included in our analyses.

### Metabolic network analysis

After exporting the Kp13-MN reconstruction, we calculated the frequency with which all compounds were involved in reactions using in-house Python scripts. Those that most frequently appeared as reaction participants were considered a potential currency compound (such as protons, water, ATP, NAD, NADH and other cofactors). After manual inspection, a total of 27 compounds were filtered out in order to avoid the creation of artificial links on the reaction graph^41^. Cytoscape v. 3.1.0 was then used for network visualization and calculation of topological metrics^42^. In this representation, nodes represent reactions and there exists an edge between two nodes if a product of a reaction is used as a substrate on the reaction that follows. Analysis of choke-points (reactions that uniquely consume or produce a given substrate or product, respectively)^43^ was conducted in order to identify potential drug targets from the metabolic perspective. Choke-point blockade may lead to the accumulation of a potentially toxic metabolite in the cell or the lack of production of an essential compound; thus, choke-point reactions have great significance in drug targeting. Betweenness centrality (BC) was also calculated for each node in Kp13-MN, and this topological metric was also used for prioritization of metabolic functions within the network. High values of BC for a reaction node indicate its participation as an important communication path, bridging different metabolic parts. For a given node *v* in Kp13-MN, BC(*v*) was calculated as

$${\rm{BC}}(v)=\sum _{s\ne v\ne t\in \mathrm{Kp13}-\mathrm{MN}}\frac{Qst(v)}{Qst},$$

where Q*st* is the number of shortest paths between nodes *s* and *t* in the network and Q*st*(*v*) is the number of shortest paths between nodes *s* and *t* using node *v* as intermediate.

### Essentiality criteria

To consider whether a gene was essential, we used a recently available large-scale study identifying essential growth genes in *K. pneumoniae* described by Ramage *et al*.^44^. Furthermore, we also analyzed an experimentally validated *in silico* genome-scale metabolic reconstruction available for *K. pneumoniae* MGH 78578 described by Liao *et al*.^45^. This work predicted 118 essential genes for this strain based on *in silico* knock-outs. Bi-directional Best Hit (BBH) criterium was used to map MGH 78578 genes onto the Kp13 genome. Based on these data sources, we assigned a given gene as essential if it was reported as such either in the Ramage *et al*. or the Liao *et al*. data.

### Non-host homologous proteins analysis and microbiome conservation

The Kp13 proteome was used as query in BLASTp against the predicted human proteome (from version GRCh38.p10) to identify non-host homologous targets. Hits with *E-value* smaller than 10^−5^ were conserved. For further target prioritization, hits with identity ≥40% with a human protein were filtered out, as they could share a high degree of structural conservation that could lead to cross-interference if the bacterial protein was used as a target. A number of organisms are known to inhabit the healthy individual’s gut. Inadvertent inhibition of proteins of the normal flora is also likely to result in adverse effects. In order to mitigate this possibility, Kp13 proteins were compared to the proteins of the gut flora sequenced by the Human Microbiome Project^46^. The full list of 226 organisms is provided in Supplementary Table S1. For each sequence present in the Kp13 proteome, we analyzed the number of organisms that present at least one significant hit (*E-value* ≤10^−5^; identity ≥40%).

### Analysis of genes conserved among pathogenic *K. pneumoniae*

Mauve^47^ was used to search for groups of orthologs among different *K. pneumoniae* proteomes (with identity ≥60% and coverage ≥70%). The conservation of a protein in multiple *K. pneumoniae* genomes implies that a drug binding such target could be used to control multiple strains of this bacterium, including from different sequence types (STs), an important trait to consider given the heterogeneity of STs circulating in different regions of the world. Thus, while we used *K. pneumoniae* subsp. *pneumoniae* str. Kp13 as a reference organism, our results can be broadly expanded to *K. pneumoniae* bacteria disseminated in various geographical regions. The complete list and genome accessions for the organisms used are available in Supplementary Table S2.

### Expression data of *K. pneumoniae* Kp13 under polymyxin B exposure

We have previously determined the transcriptional response of *K. pneumoniae* Kp13 in view of various alterations in the culture medium and in the presence of polymyxin B (PB)^48^. In here, we used this data focusing only on the expression profile of the PB-resistant bacteria compared to the control condition. Briefly, Kp13 was grown in modified Muller-Hinton broth as control. In parallel, we have induced an increased, high-level resistance to this antibiotic by growing the bacteria in solid Luria-Bertani medium (LB, Oxoid, Basingstoke, England) in the presence of crescent polymyxin B (Sigma-Aldrich, St. Louis, MO, USA) concentrations and passaging the bacteria in serial dilutions of PB beginning with a concentration of 8 μg mL^−1^ up to 64 μg mL^−1^. Before and after the induction of resistance, PB MICs were confirmed by CLSI broth microdilutions. Total RNA was extracted from cultures using RNeasy Protect Bacteria (Qiagen, USA) as per manufacturer’s instructions. cDNA sequencing was performed by Fasteris (Genève, Switzerland) on a HiSeq 2000 instrument. Bioinformatics analyses included mapping of reads to the reference Kp13 genome using Bowtie (GenBank accession nos. CP003994.1-CP004000.1), counting of reads corresponding to gene features in the annotated genome using HT-Seq^49^. and evaluating differentially expressed genes using edgeR^50^. Expression of a gene in a given condition was considered if the mean count per million yielded more than five (cpm > 5). Gene up-regulation in exposure to PB was considered during target prioritization as the corresponding protein could be related to the response against this antibiotic (considered a ‘last resort’ drug). The basis for this rationale is that these overexpressed genes and their respective proteins would be potential candidates for the development of combination therapies, since the overexpressed proteins may play a role in antibiotic inactivation. As such, their targeting would produce a synergistic effect with the antibiotic itself.

### Target prioritization pipeline

**Figure 1:**
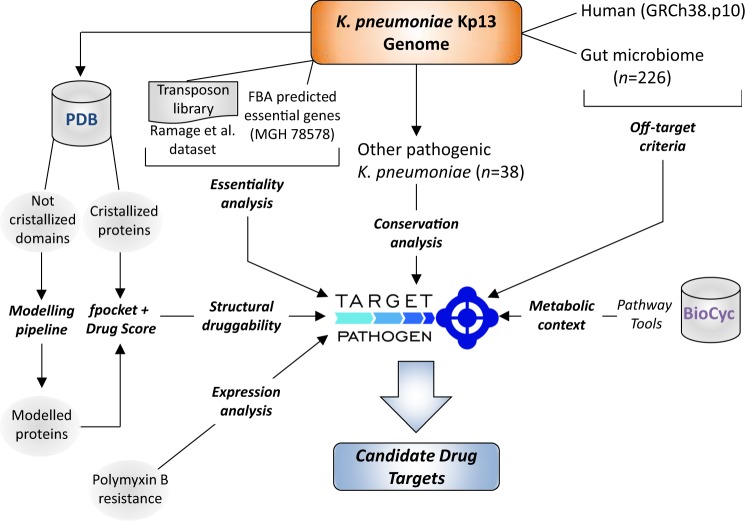
A general sketch of the prioritization pipeline. All outputs, steps, and summaries are available for download and customized analyses; see Availability of materials and data section.

All previously calculated data were integrated into Target-Pathogen (TP)^51^. TP is a platform developed by our group, which includes a database and web server for drug targets prioritization. Using this tool we obtained a ranked list of proteins with desirable features for drug targets. Firstly, we filtered out proteins with DS < 0.5 (non-druggable and poorly druggable proteins) and those that could cross-react with the human host (as per the above-stated criteria). Then, we defined three scoring functions as follows in order to assign a score to each protein in the Kp13 proteome. Equation (1) defines the importance of a protein as a target according to essentiality, conservation and metabolic context criteria, which we called ‘general targets’. Thus, for each protein we defined its score based on the following function:

1 $$SF=\frac{{E}_{mgh}+{E}_{kpn}}{2}+{C}_{v}+{C}_{y}+chk,$$

where the first term of the equation incorporates essentiality analysis as described, with ${E}_{mgh}$ and ${E}_{kpn}$ assumed to be 1 if the protein has a hit with an essential gene (as defined in Section 2.6), otherwise these terms are zeroed. ${C}_{v}\,$is the proportion of hits of the protein in different pathogenic *Kp*. ${C}_{y}\,$is the ratio between the node betweenness centrality of the associated reaction and the node with the highest centrality in Kp13-MN and $chk$ defines if the protein is associated with a chokepoint reaction ($chk=1,$ otherwise $chk=0$). Equations (2) and (3) incorporate PB resistance and commensals off-target criteria, respectively:

2 $$SF=\frac{\frac{{E}_{mgh}+{E}_{kpn}}{2}+{C}_{v}+{C}_{y}+chk}{4}+{P}_{b},$$

3 $$SF=\frac{\frac{{E}_{mgh}+{E}_{kpn}}{2}+{C}_{v}+{C}_{y}+chk}{4}-{G}_{M},$$

where ${P}_{b}$ is 1 if the protein is overexpressed in PB presence and ${G}_{M}\,$is the number of gut microbiome organisms that have at least one homologous protein in Kp13 genome, normalized by the total number of analyzed organisms. We have intentionally not set any *a priori* weights on each of the terms that compose the scoring functions in order to avoid incurring in possible representation biases, as we posited that all considered variables play an important role in defining a suitable target. A general schema of our target prioritization pipeline is shown in Fig. 1.

### Availability of materials and data

All the data generated and integrated in this study, including protein structures, metabolic annotations, essentiality information and related meta-data is openly available at the Target Pathogen^51^ web server interface. The access URL is http://target.sbg.qb.fcen.uba.ar/patho/genome/Kp13.

## Results and Discussion

### *Klebsiella pneumoniae* protein structures are enriched for druggable pockets

**Figure 2:**
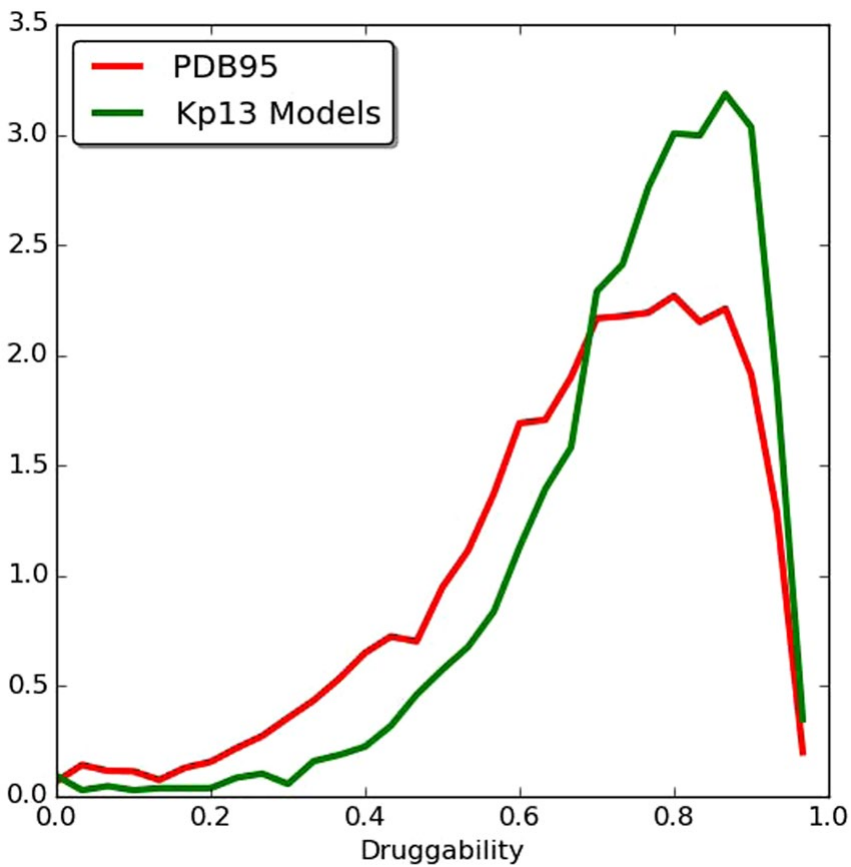
Histogram of the druggability score. All ligand-bound structures in the PDB (red line) and all the modeled structures of Kp13 (green line) are represented in the histogram. The scores were computed using the *fpocket* program for all pockets present in all unique proteins in the PDB, which were crystallized in complex with a drug-like compound. A Gaussian fit of the data made to define these sets was performed in Radusky *et al*.^33^. The sets are: non-druggable proteins (ND; DS < 0.2), poorly druggable (PD; 0.2 ≤ DS < 0.5), druggable (D; 0.5 ≤ DS < 0.7), and highly druggable (HD; DS ≥ 0.7).

Our analysis began by classifying all obtained domain structures of *K. pneumoniae* Kp13 (which included those retrieved from PDB and our own generated homology-based models) according to their structural druggability. For this, we first grouped the structural domains into four categories. The first one includes proteins from the PDB which have been experimentally obtained bound to a drug-like compound or an inhibitor (we refer to these as the Experimentally-determined Structures With Drug [ESWD] group). The second category corresponds to those proteins which structure was experimentally determined without a binding drug (Experimentally-determined Structures Without Drug group [ESWOD]). The remaining two categories include modeled structures obtained with our homology pipeline. The Modeled With Drug group (MPWD) includes models where the used template was crystallized in the presence of an inhibitor or drug-like compound. The last one includes all modeled structures that bear no relation to any structure hosting a drug-like compound (Modeled Without Drug [MPWOD]). For all structures, we computed all the possible pockets and their corresponding druggability score (DS) using *fpocket*. According to their DS, we classified all the structures in each category into four druggability groups (Table 1) (see Methods for criteria). As expected, most of the *K. pneumoniae* available structures crystallized in the presence of a drug possess high DS. For comparison, we calculated the DS for all ligand-bound structures in PDB95, a non-redundant subset of PDB, revealing an enrichment of predicted druggable pockets in the Kp13 models, as well as confirming that our method indeed produces results that are consistent in terms of detecting proteins able to host a drug-like compound (Fig. 2).

**Table 1:** *Klebsiella pneumoniae* Kp13 proteins classified according to their druggability score.

	ESWD	ESWOD	MPWD	MPWOD	Total
Non-druggable	1	27	10	30	68
Poorly druggable	14	36	57	71	178
Druggable	43	50	261	281	635
Highly druggable	115	134	874	1,190	2,313
**Total**	**173**	**247**	**1,202**	**1,572**	**3,194**

ESWD: Crystallized proteins with drugs; ESWOD: Crystallized proteins without drugs; MPWD: Proteins modeled with templates harboring a drug; MPWOD: Proteins modeled without drug.

### Reconstruction of the *K. pneumoniae* Kp13 metabolic network allows pathway contextualization of prioritized protein targets

**Figure 3:**
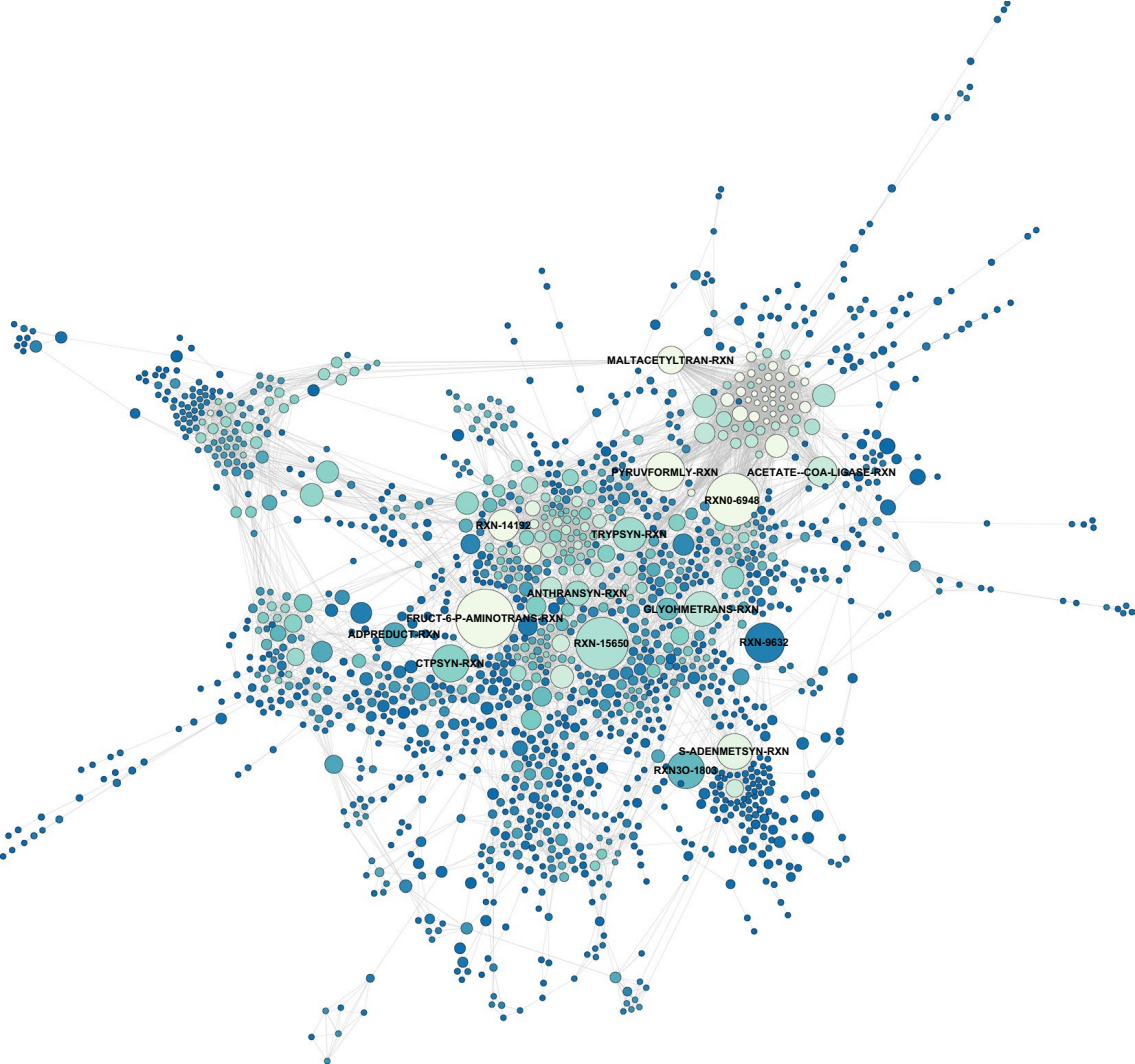
Metabolic network of *K. pneumoniae* Kp13 represented as a reaction graph. Nodes depict reactions in the network, and there exists an edge between two nodes when the product of a reaction is used as the substrate in the following reaction. Node size is proportional to betweenness centrality, and MetaCyc accessions (http://metacyc.org) for hub reactions are shown.

We performed a whole-genome-based reconstruction of the Kp13 metabolic network (Kp13-MN) using Pathway Tools algorithm and incorporating evidence from a previously curated *K. pneumoniae* metabolic network^45^, followed by manual inspection and curation of the resulting Kp13 network. Once constructed, this network was analyzed from a graph-theoretic point of view as a reaction graph, allowing the calculation of topological metrics that relate to node importance. A total of 1,969 reactions compose the Kp13-MN, with 1,847 being enzyme-catalyzed and forming part of 321 predicted metabolic pathways. 1,523 enzymes take part in these transformations. For comparison, the *Escherichia coli* K-12 substr. MG1655 metabolic network (a highly curated reconstruction available within Pathway Tools) is composed by 1,564 enzymes assigned to 1,884 reactions that further group into 339 pathways. We also identified choke-points (CPs) in the Kp13-MN, i.e. reactions that uniquely consume (input CPs) or produce (output CPs) a given compound. A total of 145 reactions were strictly classified as input CPs, while 154 reactions were strictly output CPs. On the other hand, 149 reactions were classified as CPs on both producing and consuming sides of the reaction. Mapping these CPs reactions to proteins resulted in a total of 841 proteins. Since many CPs involve the transformation of indispensable compounds, they have been proposed as attractive drug targets^43,52^. We identified that while 6% of the Kp13 proteome is composed by experimentally predicted essential proteins (from the projection of a large-scale transposon mutant library onto the Kp13 proteome)^44^, 34% of identified CPs are associated to essential proteins, a significant enrichment of almost six-fold (p-value ≤ 10^−5^, hypergeometric test) reinforcing the relevance of this parameter in our target prioritization strategy. The projection of Kp13-MN onto a reaction graph allowed the calculation of topological metrics. Particularly, betweenness centrality was used during our analyses. Figure 3 depicts the resultant Kp13-MN graph, with node sizes proportional to this metric. The presence of few high-centrality nodes indicates that these hubs may be of special importance to the cohesiveness of the network. We did not limit our analysis to solely filtering choke-point nodes or hubs identification. Rather, this information was incorporated into the scoring function that allowed ranking of the potential Kp13 targets within the proteome of this organism, which were then contextualized into metabolic subparts. The complete list of choke-points and centrality measures is also available within Target-Pathogen^51^, as well as the complete metabolic annotation of Kp13.

### Incorporation of gene essentiality, pathogen conservation, and metabolic data into the prioritization function allows identification of general targets against *Kp*

After integration of the generated multi-omic datasets, we sought to score individual proteins according to their potential as target for the control of pathogenic *Kp*. For this, two *a priori* filters were applied: firstly, proteins with orthologs in the human genome were discarded in order to minimize the chances of cross-reactivity (and toxicity) of a drug with the host protein; the second filter discarded proteins for which we could not obtain a representative structural model that harbored at least one druggable pocket. By applying these filters we obtained 2,950 candidate druggable proteins with no close homologs in the human genome (Supplementary Fig. S1). Afterward, we further ranked these proteins by taking into account different empirical features that a protein should exhibit in order to serve as an attractive target. These include its presence in related pathogens (for which the degree of conservation ultimately leads to narrow- or broad-spectrum activities), its essentiality, and the contextualization of its function into one or many metabolic pathways. When comparing all Kp13 proteins against pathogenic *Kp*, conservation revolves mainly in chromosomally-encoded features (Supplementary Fig. S2), which represents the most stable genetic element, as plasmids can be gained/lost or undergo extensive rearrangements. Based on the gathered data, we considered these parameters in a scoring function (see equation (1)) that allowed a first ranking of *Kp* proteins, a set of candidates which we called ‘general targets’. The 15 highest-ranking proteins, along with their druggability features, are presented in Table 2. This analysis can also be replicated and customized through the web interface of Target-Pathogen (see URL and reference in Availability of materials and data section).

**Table 2:** List of prioritized protein targets considering gene essentiality, pathogenic *Kp* scope and metabolic network metrics (ranked according to equation (1)).

Rank	Locus number^	Gene	Product	Product size (aa)	Structural druggability*	Essential	Choke-point	Network centrality	Presence in pathogenic *Kp* (%)	Pathways involved^#^	DrugBank Inhibitor^&^
1	01032	*fabB*	3-oxoacyl-[acyl-carrier-protein] synthase 1	407	0.74	Yes	Yes	0.64	100.0	Biotin [B], Fatty acids [B]	Approved (DB01034)
2	02296	*argA*	Amino-acid acetyltransferase	443	0.75	Yes	Yes	0	100.0	L-arginine [B]	ND
3	01798	*lpxA*	UDP-N-acetylglucosamine O-acyltransferase	263	0.88	Yes	Yes	0.29	100.0	Lipopolysaccharide [B]	Experimental (DB08558)
4	01899	*lpxC*	UDP-3-O-[3-hydroxymyristoyl] N-acetylglucosamine deacetylase	306	0.78	Yes	Yes	0.29	100.0	Lipopolysaccharide [B]	Experimental (DB07861)
5	04921	*fabH*	3-oxoacyl-[acyl-carrier-protein] synthase 3	318	0.77	No	Yes	0.64	100.0	Biotin [B], Fatty acids [B]	Approved (DB01034) Cerulenin
6	01814	*dapD*	2,3,4,5-tetrahydropyridine-2,6-dicarboxylate N-succinyltransferase	274	0.96	Yes	Yes	0.08	100.0	L-lysine [B]	Experimental (DB01856)
7	01909	*murF*	UDP-N-acetylmuramoyl-tripeptide–D-alanyl-D-alanine ligase	452	0.75	Yes	Yes	0.08	100.0	Peptidoglycan [B]	Experimental (DB06970)
8	03831	*dapE*	succinyl-diaminopimelate desuccinylase	375	0.81	Yes	Yes	0.08	100.0	L-lysine [B]	ND
9	01800	*lpxD*	UDP-3-O-[3-hydroxymyristoyl] glucosamine N-acyltransferase	341	0.82	Yes	Yes	0.07	100.0	Lipopolysaccharide [B]	ND
10	05433	*fabI*	Enoyl-[acyl-carrier-protein] reductase [NADH]	262	0.72	Yes	Yes	0.06	100.0	Biotin [B], Fatty acids [B]	Approved (DB08604) Triclosan
11	01797	*lpxB*	Lipid-A-disaccharide synthase	383	0.92	Yes	Yes	0.06	100.0	Lipopolysaccharide [B]	ND
12	01905	*murG*	undecaprenyl-PP-MurNAc-pentapeptide-UDPGlcNAc GlcNAc transferase	356	0.80	Yes	Yes	0.06	100.0	Peptidoglycan [B]	Murgocil (Ref.^106^)
13	00662	*asd*	Aspartate-semialdehyde dehydrogenase	368	0.58	Yes	Yes	0.05	100.0	L-lysine [B], L-threonine [B], L-methionine [B], L-homoserine [B]	Experimental (DB03502)
14	04866	*purB*	Adenylosuccinate lyase	456	0.84	Yes	Yes	0.05	100.0	Purine nucleotides [B]	ND
15	04914	*tmk*	Thymidylate kinase	213	0.58	Yes	Yes	0.05	100.0	Pyrimidine deoxyribonucleotides [B]	Experimental (DB03280)

^^^The *K. pneumoniae* Kp13 locus suffix ‘KP13_’ is omitted; *druggability of the protein considering the highest scoring pocket. ^#^B: biosynthesis. ^&^Data gathered from http://www.drugbank.ca for orthologs of each protein with relevance studied on a per-target basis. ND, not determined.

**Figure 4:**
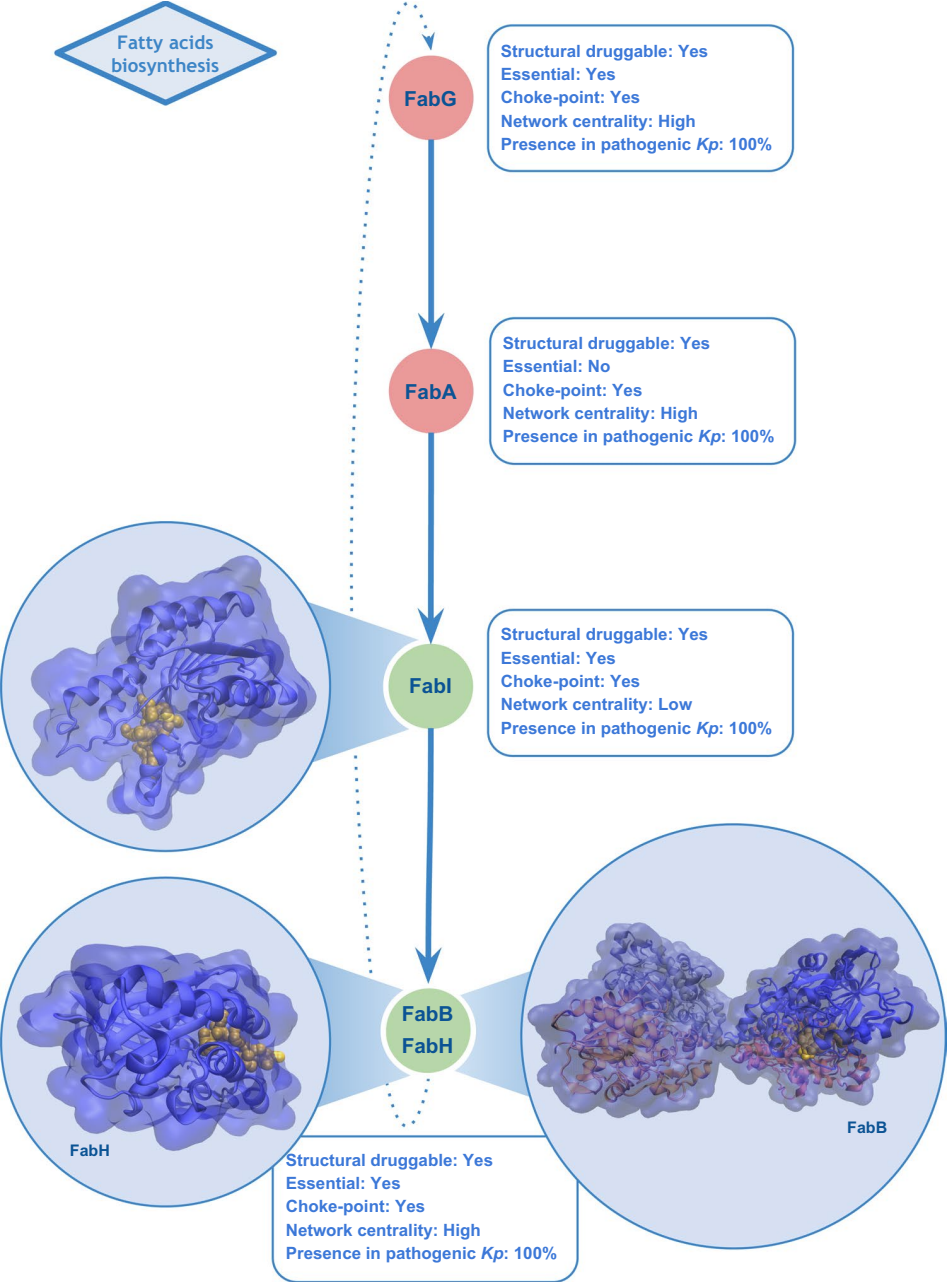
A subset of the fatty acid elongation pathway. Structures correspond to FabB, FabI and FabH, three among the 15 top-ranked candidates in the scoring pipeline for drug target selection in *Kp*. Representation of the most druggable pocket is shown in yellow within the structures. Green nodes indicate proteins that were top-ranked in our analyses. Conservation (in percent) of each protein in 38 pathogenic *Kp* genomes is also shown.

**Figure 5:**
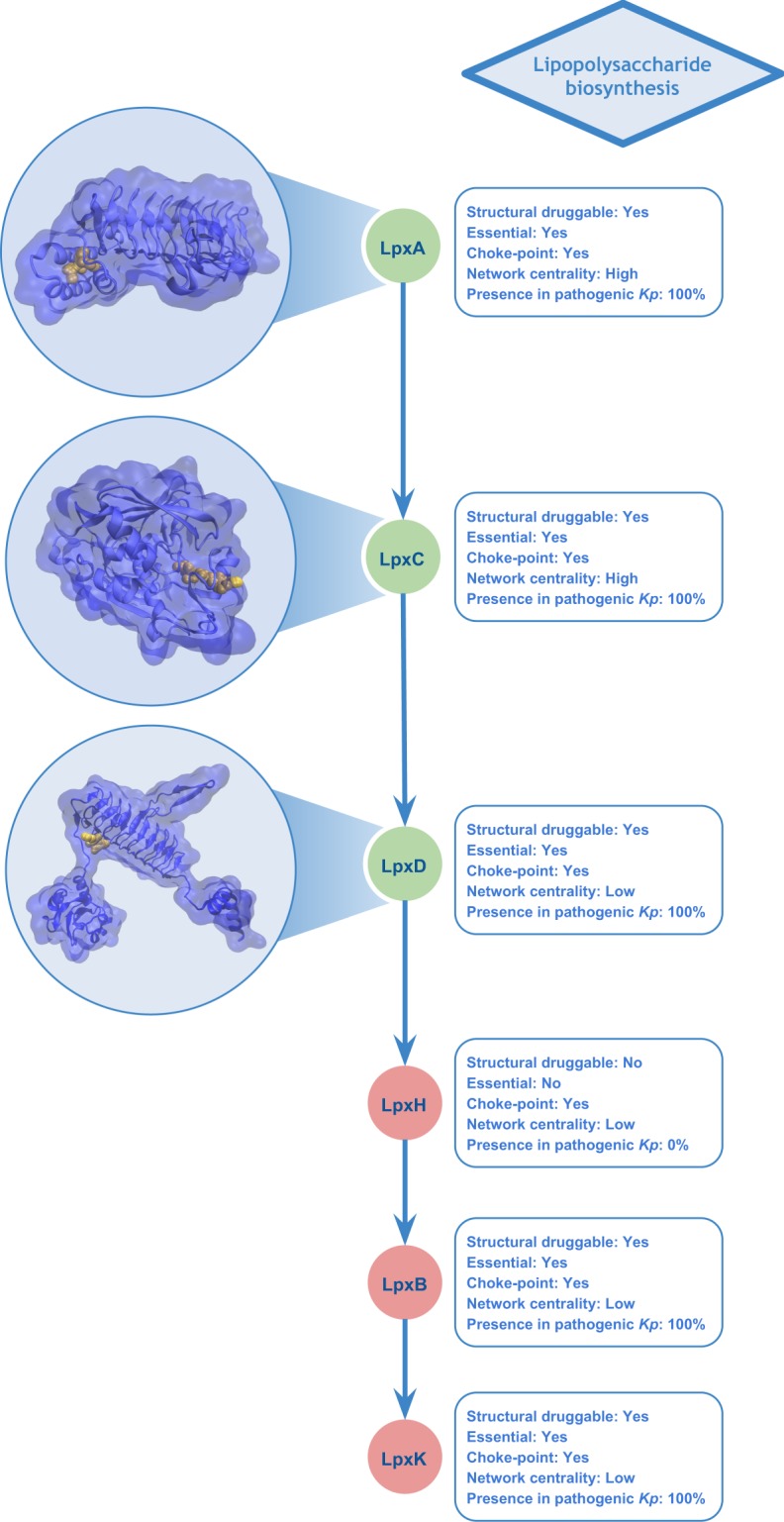
Lipid IV_A_ biosynthesis, an attractive metabolic pathway for drug targeting. Structures correspond to candidate target proteins, LpxA, LpxC and LpxD. Representation of the most druggable pockets is shown in yellow within the structures. Green nodes indicate proteins that were top-ranked in our analyses. Conservation (in percent) of each protein in 38 pathogenic *Kp* genomes is also shown.

Among the shortlisted candidates, it is interesting to note metabolic pathway activities that are currently targeted by antimicrobials, a positive indicator that the data generated and integrated using our methodology allows the recovery and contextualization of biologically relevant metrics that can be used as a proxy for enriching candidate proteins with desirable characteristics for the control of *Kp*. The pathways identified included fatty acids, lipopolysaccharide (LPS), peptidoglycan, pyrimidine deoxyribonucleotides and purine nucleotides biosynthesis. Proteins involved in fatty acid biosynthesis components pathways (e.g. 3-oxoacyl-[acyl-carrier-protein] synthase 1 and 3 and enoyl-[acyl-carrier-protein] reductase [NADH]) are druggable, essential, conserved and majorly related to important reactions from the metabolic point of view and principally are choke-points (Fig. 4). This pathway allows homeostasis of the bacterial membrane^53,54^. Particularly, the enoyl-[acyl-carrier-protein] reductase [NADH] (FabI) has been targeted for development of new antibacterial agents^55,56^. Synthesis of lipopolysaccharide (LPS), an essential component of the Gram-negative outer membrane, also appeared top-ranked, with cytoplasmic enzymes LpxA, LpxC and LpxD involved in the initial steps of lipid A production through the Raetz pathway^57^. They also fulfil most of the above-defined criteria that make a protein attractive for drug targeting (Fig. 5). In the past two decades numerous LpxC inhibitors have been developed as bactericidal agents against pathogenic Gram-negative organisms including *K. pneumoniae*, with recent comprehensive reviews detailing these developments^58,59^. However, only a single molecule (ACHN-975) entered human clinical trials, being later discontinued during Phase I due to unwanted inflammatory effects at the injection site^60^. However, the research for LpxC inhibitors has not been discontinued and recently a novel inhibitor promising to be of value for clinical development (LPC-069, a biphenylacetylene-based LpxC inhibitor) was proposed to combat a broad panel of Gram-negative clinical isolates, including several multiresistant and extremely drug-resistant strains with no known adverse effects in mice^61^. Accordingly, our results showed that the gene encoding LpxC protein is conserved in all studied pathogenic strains of *K. pneumoniae* and does not present close homologs within the human proteome.

### Incorporation of a polymyxin B overexpression term allows identification of drug-related targets in resistant *Kp*

Once targets that complied with rules associated with gene essentiality, metabolic importance and broad *Kp* conservation were identified, we incorporated in our scoring function a term related to overexpression during polymyxin B (PB) exposure. PB is an antibiotic considered as ‘last resort’ in the treatment of infections caused by CRE pathogens. We reasoned that *Kp* proteins that are overexpressed when exposed to the antibiotic may have a role in counteracting the deleterious effects of the drug in the bacterial cell, as was shown for other pathogens^62^. Further, these proteins need not be necessarily resistance-related or involved in alterations to outer membrane components. Notably, polymyxins have been shown to induce rapid killing at concentrations considerably lower than that required for cytoplasmic membrane permeabilization or depolarization, which suggest that other bactericidal effects may be involved^63^. We have previously shown that the gene expression response elicited by PB treatment in *Kp* affects a myriad of transcriptional regulators such as two-component systems, which in turn impact the expression of a broad and diverse set of genes^48^. In this report, we also showed that PB induces, along with alterations of genes involved in the biosynthesis of outer membrane components, various metabolic shifts in *K. pneumoniae*^48^, which are in the same line of earlier evidence showing that this compound also has intracellular enzymatic targets^64^. In this sense, the targets identified using this strategy could also be of interest in a combination therapy perspective when dealing with resistant *Kp* infections, possibly acting synergistically with other drugs, in a fashion involving a non-antimicrobial with a bactericidal compound. As proof of this concept, in other infectious diseases, such as bacteremia caused by *Pseudomonas aeruginosa*, the combination of efflux proteins inhibitors (such as phenyl-arginine-β-naphthylamide) and iron chelators have been proposed to control the infection process in view of the overexpression of the MexAB-OprM efflux system during iron deprivation^65^. Table 3 presents the list of protein targets resulting from this analysis.

**Table 3:** List of prioritized protein targets by incorporating protein overexpression in PB exposure (ranked according to equation (2)).

Rank	Locus number^	Gene	Product	Product size (aa)	Structural druggability*	Essential	Choke-point	Network centrality	Presence in pathogenic *Kp* (%)	Pathways involved^#^	DrugBank Inhibitor^&^
1	01032	*fabB*	3-oxoacyl-[acyl-carrier-protein] synthase 1	407	0.76	Yes	Yes	0.64	100.0	Biotin [B], Fatty acids [B]	Approved (DB01034)
2	01798	*lpxA*	UDP-N-acetylglucosamine O-acyltransferase	263	0.88	Yes	Yes	0.29	100.0	Lipopolysaccharide [B]	Experimental (DB08558)
3	01899	*lpxC*	UDP-3-O-[3-hydroxymyristoyl] N-acetylglucosamine deacetylase	306	0.78	Yes	Yes	0.29	100.0	Lipopolysaccharide [B]	Experimental (DB07861)
4	01814	*dapD*	2,3,4,5-tetrahydropyridine-2,6-dicarboxylate N-succinyltransferase	274	0.96	Yes	Yes	0.08	100.0	L-lysine [B]	Experimental (DB01856)
5	01800	*lpxD*	UDP-3-O-[3-hydroxymyristoyl] glucosamine N-acyltransferase	341	0.82	Yes	Yes	0.07	100.0	Lipopolysaccharide [B]	ND
6	00662	*asd*	Aspartate-semialdehyde dehydrogenase	368	0.58	Yes	Yes	0.05	100.0	L-lysine [B], L-threonine [B], L-methionine [B], L-homoserine [B]	Experimental (DB03502)
7	03824	*dapA*	Dihydrodipicolinate synthase	292	0.81	Yes	Yes	0.03	100.0	L-lysine [B]	Experimental (DB02370)
8	01459	*kdsA*	2-dehydro-3-deoxyphosphooctonate aldolase	284	0.71	Yes	Yes	0.03	100.0	3-deoxy-D-manno-octulosonate [B], lipopolysaccharide [B]	Experimental (DB02433)
9	00796	*pssA*	CDP-diacylglycerol–serine O-phosphatidyltransferase	451	0.86	Yes	Yes	0.06	97	Phosphatidylethanolamine [B]	ND
10	01108	*glmM*	Phosphoglucosamine mutase	445	0.54	Yes	Yes	0.01	100.0	UDP-N-acetyl glucosamine [B]	ND
11	31485	*fabD*	Malonyl CoA-acyl carrier protein transacylase	309	0.70	Yes	Yes	0.0	100.0	Fatty acids [B]	ND
12	00029	*glmS*	Glucosamine–fructose-6-phosphate aminotransferase isomerizing	609	0.91	Yes	Yes	1.0	100.0	UDP-N-acetyl-D-glucosamine [B], O-antigen [B]	Experimental (DB02445)
13	04702	*galU*	UTP–glucose-1-phosphate uridylyltransferase	300	0.57	Yes	Yes	0.17	100.0	UDP-glucose [B]	ND
14	01466	*ispE*	4-diphosphocytidyl-2-C-methyl-D-erythritol kinase	283	0.55	Yes	Yes	0.12	100.0	Isoprenoid [B]	Experimental (DB03687)
15	03868	*fabA*	3-hydroxydecanoyl-[acyl-carrier-protein] dehydratase	188	0.93	Yes	Yes	0.10	100.0	Biotin [B], Fatty acids [B]	Experimental (DB03813)

^The *K. pneumoniae* Kp13 locus suffix ‘KP13_’ is omitted; ^*^druggability of the protein considering the highest scoring pocket. ^#^B: biosynthesis. ^&^Data gathered from http://www.drugbank.ca for orthologs of each protein with relevance studied on a per-target basis. ND, not determined.

Of notice, many of the metabolic roles involving the identified targets are also important to cellular homeostasis, such as L-lysine (performed by tetrahydropicolinate succinylase, dihydrodipicolinate synthase and aspartate-semialdehyde dehydrogenase) and isoprenoid biosynthesis through 2C-methyl-D-erythritol 4-phosphate (MEP) pathway (by the intermediate CDP-ME kinase IspE), besides other previously detected metabolic roles such as fatty acid biosynthesis (3-oxoacyl-[acyl-carrier-protein] synthase 1; 3-hydroxydecanoyl-[acyl-carrier-protein] dehydratase; malonyl CoA-acyl carrier protein transacylase) and membrane components metabolism (LpxA, LpxC, LpxD, GalU, KdsA, GlmM, GlmS, PssA). For several of these protein targets, some inhibitors have already been determined, as shown in Table 3. In the following, we discuss some aspects of the candidate proteins IspE and PssA, which are attractive either in monotherapy or in polymyxin combination therapy.

IspE is a cytoplasmic kinase of the MEP pathway that is involved in the biosynthesis of the isoprenoids used by many Gram-negative bacteria (including *E. coli, Salmonella enterica, P. aeruginosa* and *H. influenzae*), as well as Gram-positive bacteria such as *Clostridium difficile* and *Bacillus subtilis*, *M. tuberculosis*, and even few apicoplast protozoa such as *Plasmodium falciparum*^66^. Because isoprenoids are involved in a wide variety of vital biological functions, the seven enzymes without close human homologs that participate in their metabolism (Dxs, IspC, IspD, IspE, IspF, IspG, IspH) are favorable candidate drug targets and several inhibitors have been already reported^67^, mainly as antimalarial targets^68,69^. In Gram-negative bacteria, compounds from the isoxazol-5(4 H)-one series have been evaluated (e.g. PubChem compound ID 3768522 and DrugBank DB03687, Table 3) showing inhibitory activities against IspE/Ipk from *E. coli* and *Y. pestis*^70^, and the study of its effect in *Klebsiella* bacteria is also appealing in the light of our results.

The active form of phosphatidylserine (PtdSer) synthase (PssA) from some bacteria is a cytoplasmic membrane-associated enzyme that converts cytidine diphosphate diacylglycerol (CDP-DAG) and serine (L-Ser) to PtdSer, a negatively charged phospholipid that is rapidly decarboxylated by PtdSer decarboxylase (Psd) to generate phosphatidylethanolamine (PtdEtn), the major phospholipid of membranes^71^. PssA has been shown to play a significant role in virulence of *Brucella abortus* in a mouse model of infection with a Δ*pssA* mutant^72^. Interestingly, the *Brucella* cell envelope is normally resistant to the bactericidal action of polycationic peptides such as PB, but the Δ*pssA* mutant of *B. abortus* showed loss of PtdEtn and increased sensitivity to this drug, without any changes in its LPS structure^72^. Thus, an evaluation of a combination of PB and an inhibitor against PssA seems as a plausible approach. Polymyxin combination therapy is engaging since it has been reported to increase bacterial killing and reduce the development of polymyxin-resistant subpopulations^73^, even in multidrug-resistant *K. pneumoniae* isolates^74^. This type of polytherapy may enhance bacterial killing via subpopulation and/or mechanistic synergy^75^. Indeed, *in vitro* tests combining PB and the carbapenem tigecycline using a hollow-fiber infection model have shown a bactericidal effect against CRE while suppressing the emergence of PB resistance^76^. Also, a combination of PB with a non-antibiotic drug like selective estrogen receptor modulators (SERMs) demonstrated excellent antibacterial killing kinetics against polymyxin-resistant *P. aeruginosa, A. baumannii*, and *K. pneumoniae*^77^. Given the benefit of polymyxin combination therapy, future studies could be performed to validate the activity of other polymyxin-based combinations, such as those described hereinafter.

Comparing these results with the general targets set (Table 2), there was an overlap of six candidate targets, which was not unexpected since the scoring function used to rank both lists differed by a single parameter (*P*_*b*_ term in Eq. 2) related to overexpression in PB, although it corresponded to half of the total contribution to the ranking scheme. It is interesting to see the presence of proteins related to highly central reactions in the Kp13-MN such as glucosamine–fructose-6-phosphate aminotransferase isomerizing (GlmS; reaction betweenness centrality = 1.0) and 3-oxoacyl-[acyl-carrier-protein] synthase (FabB; reaction betweenness centrality = 0.64). When considering structural druggability as predicted by *fpocket*, eleven out of the 15 top-ranked proteins have at least one pocket considered highly druggable (DS ≥ 0.7).

### Gut microbiota conservation allows prioritization of protein targets less likely to interfere with commensal gut bacteria

As the last step in our *Kp* target prioritization pipeline, we sought to identify candidate proteins for which developed drugs would present enhanced selectivity towards bacterial pathogens, thus minimizing the impact to the commensal gut microbiota. This was achieved by modifying the scoring function in order to include a term that penalizes the score of a protein in up to 50% with the increasing presence of orthologs in gut commensal species (see equation (3)). A total of 803,381 proteins sequences present in the compared proteomes were used in this analysis. Table 4 presents the 15 top-ranked targets taking this concept into account. These prioritized proteins are variably present, albeit in low frequency, among the 226 commensal genomes, with their occurrence ranging from 0.4–22%. As a comparison, current antibiotics targets such as DNA gyrase (*gyrA* [KP13_00955]), targeted by fluoroquinolones, and RNA polymerase (*rpoB* [KP13_01360]), target of rifamycins, are present in 99.6% and 99.1%, respectively, of the gut genomes using the same criteria (Supplementary Table S3).

**Table 4:** List of prioritized protein targets by incorporating protein conservation among gut microbiomes (ranked according to equation (3)).

Rank	Locus number^	Gene	Product	Product size (aa)	Structural druggability*	Essential	Choke-point	Network centrality	Presence in pathogenic *Kp* (%)	Pathways involved^#^	DrugBank Inhibitor^&^
1	02296	*argA*	Amino-acid acetyltransferase	443	0.75	Yes	Yes	0.89	100	L-ornithine [B], L-arginine [B]	ND
2	01032	*fabB*	3-oxoacyl-[acyl-carrier-protein] synthase 1	407	0.74	Yes	Yes	0.64	100	Biotin [B], Fatty acids [B]	Approved (DB01034)
3	00796	*pssA*	CDP-diacylglycerol–serine O-phosphatidyltransferase	451	0.86	Yes	Yes	0.06	97	Phosphatidylethanolamine [B]	ND
4	01800	*lpxD*	UDP-3-O-[3-hydroxymyristoyl] glucosamine N-acyltransferase	341	0.82	Yes	Yes	0.07	100	Lipopolysaccharide [B]	ND
5	01909	*murF*	UDP-N-acetylmuramoyl-tripeptide–D-alanyl-D-alanine ligase	452	0.76	Yes	Yes	0.08	100	Peptidoglycan [B]	Experimental (DB06970)
6	01907	*murD*	UDP-N-acetylmuramoylalanine–D-glutamate ligase	438	0.71	Yes	Yes	0.02	100	Peptidoglycan [B]	Experimental (DB03801)
7	01797	*lpxB*	Lipid-A-disaccharide synthase	383	0.92	Yes	Yes	0.06	100	Lipopolysaccharide [B]	ND
8	05359	*trpA*	Tryptophan synthase alpha chain	270	0.63	Yes	Yes	0.52	100	L-tryptophan [B]	ND
9	00662	*asd*	Aspartate-semialdehyde dehydrogenase	368	0.58	Yes	Yes	0.05	100	L-lysine [B], L-threonine [B], L-methionine [B], L-homoserine [B]	Experimental (DB03502)
10	01899	*lpxC*	UDP-3-O-[3-hydroxymyristoyl] N-acetylglucosamine deacetylase	306	0.78	Yes	Yes	0.29	100	Lipopolysaccharide [B]	Experimental (DB07861)
11	04194	*lpxK*	Tetraacyldisaccharide 4′-kinase	326	0.69	Yes	Yes	0	100	Lipopolysaccharide [B]	ND
12	01905	*murG*	undecaprenyl-PP-MurNAc-pentapeptide-UDPGlcNAc GlcNAc transferase	356	0.8	Yes	Yes	0.06	100	Peptidoglycan [B]	Murgocil (ref.^106^)
13	31828	*murE*	UDP-N-acetylmuramoyl-L-alanyl-D-glutamate–2,6-diaminopimelate ligase	495	0.85	Yes	Yes	0.03	97	Peptidoglycan [B]	Experimental (DB03801)
14	05226	*hpxT*	5-hydroxyisourate hydrolase	109	0.6	Yes	Yes	0.007	100	Purine metabolism	ND
15	03831	*dapE*	Succinyl-diaminopimelate desuccinylase	375	0.81	Yes	Yes	0.08	100	L-lysine [B]	ND

^The *K. pneumoniae* Kp13 locus suffix ‘KP13_’ is omitted; ^*^druggability of the protein considering the highest scoring pocket. ^#^B: biosynthesis. ^&^Data gathered from http://www.drugbank.ca for orthologs of each protein with relevance studied on a per-target basis. ND, not determined.

**Figure 6:**
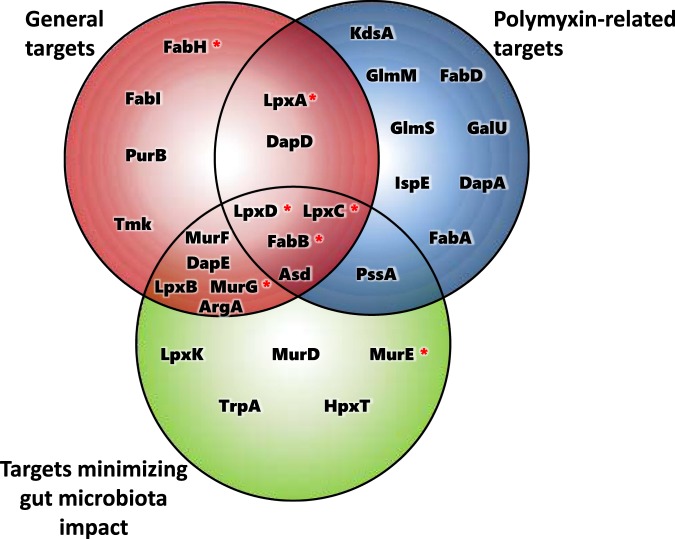
Venn diagram showing the number of unique and shared targets identified using the three different ranking strategies for drug targeting. Targets that have been experimentally tested with inhibitors in *K. pneumoniae* are marked with an asterisk: LpxA^101,102^, LpxD^101^, FabB/FabH^103^, LpxC^104^, and MurG/MurE^105^.

Our strategy of ranking protein targets in pathogenic *Kp* while minimizing possible deleterious effects to the commensal microbiome led to the identification, among the top 15 highest-ranking proteins, of four cytoplasmic candidates (FabB, LpxC, LpxD, Asd) that we had previously identified using the first two approaches (Fig. 6), and could thus be considered as high-value targets. These represent proteins that comply not only with our employed structural druggability criterium (presenting DS ≥ 0.7, highly druggable), but that also possess features desirable from the genomic point of view, such as being classified as essential and conserved in all pathogenic *Kp* considered. They are also important from a metabolic perspective being classified as choke-points in the Kp13-MN; one of them (FabB) is also related to a high-centrality reaction in the network (0.64 normalized betweenness centrality). These proteins participate in fundamental processes of the Kp13-MN, including LPS biosynthesis (LpxC, LpxD), biotin and fatty acids production (FabB). The candidate targets shortlisted in Tables 2, 3 and 4 and their interrelations (Fig. 6) comprise twenty-nine unique proteins with characteristics that are desirable from a druggability perspective, and their follow-up could be promising given the need of novel drugs developed for controlling infections caused by resistant bacteria. In this sense, proteins that are high-ranking all display interesting features that could be exploited in future drug development works. For instance, the integrated analysis of the 29 unique candidates reveals the emergence of a core metabolic subset shared between many of them, comprising biosynthesis of amino acids, fatty acids, and cell wall components. These represent fundamental metabolic aspects of the bacterial cellular machinery, and the specific proteins identified here represent a good starting point for further experimental exploration as well as confirmation of their relevance in ongoing efforts against some of these.

### Beyond the upper rank: assessing intermediate-value targets

In this section, we explore candidates that, although did not rank in the Top 15 in any of the three previous ranking strategies, present biological evidence that would be suitable from a druggability perspective. We refer to these candidates as intermediate-value targets. These were identified by studying the ranked list of 100 candidates beyond the previously discussed top-ranked in all three equations. For instance, intermediate-value targets common between the three equations that are druggable, essential, highly conserved in pathogenic *Kp*, overexpressed in PB, metabolic choke-points and have low microbiome representation (around 10%) are MurI (KP13_31500, glutamate racemase) and cytoplasmic membrane protein FtsI (KP13_01911, peptidoglycan synthase FtsI/PBP-3) (Supplementary Table S3). These targets could be attractive in a polymyxin combination perspective addressing drug resistance in early stages of antimicrobial drug discovery. Also, these protein targets participate in peptidoglycan biosynthesis, and targets directed towards them would have a broad Gram-positive and Gram-negative spectrum, which is the case for the third-generation cephalosporin cefdinir (DrugBank ID DB00535), the cognate inhibitor of FtsI (PBP3). We did not find reports in the literature of using polymyxin B or colistin in combination therapy with cefdinir for the treatment of MDR infections. For glutamate racemase (MurI) experimental inhibitors are under investigation in *Helicobacter pylori* (DrugBank ID DB08698), *Listeria monocytogenes* (DrugBank ID DB02343), *Enterococcus faecalis* (DrugBank ID DB07937) and *Streptococcus pyogenes* (DrugBank ID DB08272).

Protein targets involved in the folate biosynthesis pathway were also ranked as intermediate-value targets, and include the cytoplasmic protein FolE (GTP cyclohydrolase 1), which is druggable, essential, highly conserved in pathogenic *Kp*, overexpressed in PB, choke-point and have intermediate microbiome representation (25%), as well as the cytoplasmic protein FolB (dihydroneopterin aldolase), which although not overexpressed in PB is druggable, essential, highly conserved, choke-point and presents low microbiome representation (8%). Structural studies with type IB GTP cyclohydrolase 1 (GCYH-IB) enzyme from *N. gonorrhoeae* showed that GCYH-IB exhibits marked differences in binding to its analog substrate compared to the canonical type IA GTP cyclohydrolase 1 involved in biopterin biosynthesis in human and other animals^78^. These structural differences could be exploited in the design of inhibitors specific for GCYH-IB. With respect to FolB, it has recently been demonstrated in *M. tuberculosis* that the gene essentiality lies in the aldolase and/or epimerase activities of the enzyme^79^, and efforts to develop inhibitors of these activities should be further pursued.

Holo-[acyl-carrier-protein] synthase (ACPS) have an essential role mediating the transfer of acyl fatty acid intermediates during the biosynthesis of fatty acids and phospholipids^80^. This cytoplasmic enzyme was classified as intermediate target, being druggable, essential, highly conserved, choke-point and with poor microbiome representation (12.4%). Interestingly, ACPS enzymes from Gram-negative and Gram-positive bacteria and *Mycoplasma pneumoniae* exhibit different native structures and substrate specificities^81^, which could turn ACPS into a narrow-spectrum target. Recently, a detailed characterization of ACPS from *E. coli* was performed and the results showed that it forms a trimer, which is structurally different to that of human ACPS, a single polypeptide that folds into an intramolecular dimer^82,83^. By exploring these differences it will be possible to find specific inhibitors against prokaryotic ACPS enzymes.

Another candidate for prioritization is the cytoplasmic enzyme glutamate–cysteine ligase (GshA, KP13_02611), which appeared overexpressed in PB, is conserved among all pathogenic *Kp* and lowly present (8.4%) in the microbiome genomes, while also being a metabolic choke-point (Supplementary Table S3). GshA plays a role in the synthesis of glutathione, a thiol-type compound that can counter the toxic actions of reactive oxygen species (ROS) and other deleterious substances, thus maintaining an intracellular reducing environment. These can be produced by the host as a response to the infection process as well as during general stress conditions including antibiotic exposure. A proof-of-principle for the targeting of bacterial thiol‐dependent antioxidant systems has successfully shown the plausibility of this strategy to combat infections caused by MDR Gram‐negative bacteria^84^, and the use of combination of drugs capable of disrupting such detoxification systems as well as exerting a bactericidal action would help in sensitizing bacteria to oxidative stress and possibly improve the bacterial killing capacity.

Finally, the analysis of intermediate-value targets identified a series of enzymes which possess kinase activities. These also represent promising candidates for further pursuit given that kinase inhibitors are among the most successful drugs developed, with protein-kinases representing the largest group of targets after G-protein coupled receptors, although the overwhelming majority of these inhibitors are directed towards human enzymes^85^. Recent efforts have shown that inhibitors of human kinases could be repurposed for use against bacterial enzymes in a combination strategy and identified that the *Listeria monocytogenes* Penicillin-binding-protein And Serine/Threonine kinase-Associated (PASTA) kinase PrkA could be inhibited by GSK690693, an imidazopyridine aminofurazan-type kinase inhibitor^86^. The list of intermediate-value targets includes ten kinases with potential for further experimental evaluation, as some of them have attractive features from the drug development perspective (Table 5). For instance, the cytoplasmic protein N-acetylglutamate (NAG) kinase (ArgB), which promotes phosphorylation of NAG in a rate-limiting step of bacterial L-arginine production, occurs through acetylated intermediates, unlike mammals which use non-acetylated intermediates, and for this reason was previously considered a candidate drug target^87^. The cytoplasmic protein FolK, along with previously discussed FolB and FolE, also participates in the folate pathway, which is already targeted by trimethoprim, an inhibitor of dihydrofolate reductase, although resistance to this drug is on the rise^88^. Positively, *folK* was found as essential in a transposon mutant library reported by Ramage *et al*.^44^ and appeared as a choke-point in our metabolic reconstruction. Another interesting candidate is thiamine-monophosphate kinase (*thiL*), which was also found as essential in the Ramage *et al*. study and participates in the synthesis of thiamine diphosphate, which is a cofactor of several key enzymes including pyruvate dehydrogenase, ∝-ketoglutarate dehydrogenase, and acetolactate synthase^89^. Taken together, these results point to a plausible role of proteins at the intermediate rank positions as candidate targets for further prioritization to the control of *Kp*, particularly due to their relevant biological roles.

**Table 5:** List of kinases identified as intermediate-value targets and their druggability features.

Gene	Kp13 Locus ID^	Product	Druggability features	Subcellular localization
*anmK*	05161	Anhydro-N-acetylmuramic acid kinase	CP, PB overexpressed, Patho*Kp*: 100%, Microbiome: 12.8%	Cytoplasmic Membrane
*argB*	00555	Acetylglutamate kinase	CP, Essential, Patho*Kp*: 100%, Microbiome: 8.4%	Cytoplasmic
*bglK*	03813	Beta-glucoside kinase	CP, Patho*Kp*: 97.4%, Microbiome: 1.3%	Cytoplasmic
*folK*	01855	2-amino-4-hydroxy-6-hydroxymethyldihydropteridine pyrophosphokinase	CP, Essential, Patho*Kp*: 100%, Microbiome: 33.2%	Cytoplasmic
*iolC*	01316	5-dehydro-2-deoxygluconokinase	CP, PB overexpressed, Patho*Kp*: 100%, Microbiome: 4.9%	Cytoplasmic
*lysC*	00360	Lysine-sensitive aspartokinase 3	CP, PB overexpressed, Patho*Kp*: 100%, Microbiome: 8%	Cytoplasmic
*mak*	02078	Fructokinase	CP, PB overexpressed, Patho*Kp*: 100%, Microbiome: 7.1%	Cytoplasmic
*selD*	05414	Selenide, water dikinase	CP, PB overexpressed, Patho*Kp*: 100%, Microbiome: 21.2%	Cytoplasmic
*thiL*	02043	Thiamine-monophosphate kinase	CP, Essential, Patho*Kp*: 100%, Microbiome: 11.5%	Unknown
*thrB*	01992	Homoserine kinase	CP, Essential, Patho*Kp*: 100%, Microbiome: 8%	Cytoplasmic

^The *K. pneumoniae* Kp13 locus suffix ‘KP13_’ is omitted. CP, choke-point; PB, polymyxin B; patho*Kp*. % conservation in 38 pathogenic *K. pneumoniae*; Microbiome, % conservation in 226 gut genomes.

## Concluding Remarks

We developed and applied an integrative analysis framework for the prioritization of protein targets using as model organism *Klebsiella pneumoniae* strain Kp13, a multidrug-resistant (including polymyxin) bacterium responsible for nosocomial infections. Various layers of information were combined, including whole-structural, metabolic, genomic and expression data as input to scoring functions that allowed a shortlisting of targets with desirable characteristics from a druggability standpoint. Out of 5,736 predicted proteins that form the proteome of Kp13, we obtained structural models for 3,194 of them and predicted the presence and location of pockets that were characterized by their druggability. The reconstruction and annotation of the metabolic network of this strain allowed the identification of the metabolic complement and enzymatic activities performed by Kp13 and related bacteria, as well as important topological metrics in this network. This was used to contextualize the functional aspects of the candidate targets identified. All this information, along with other genomic features calculated for each protein, were loaded into an openly available web server^51^ (see Availability of materials and data section), that allows easy retrieval of any of the generated data, along with parameter customization.

By applying three distinct scoring schemes, each focused on one specific aspect of druggability (1 - targets that could be viewed as having a broad importance; 2 - targets that relate to PB resistance; and 3 - targets minimizing impact towards the gut microbiome), we were able to delineate an unique set of 29 proteins with no close homologues in the human genome and that are of interest to the scientific community. The finding that some of these proteins have already been proposed, or are currently being used, as drug targets is positive evidence that our methodology allowed the selection of biologically relevant candidates. For instance, LpxC, involved in the first two steps of lipid A biosynthesis (Fig. 5), was highly ranked in all strategies. Given the lack of this compound in mammals (non-host homology) and the importance of lipid A to the stabilization of the Gram-negative bacterial membrane (broad-spectrum importance and essentiality), this pathway has been proposed as attractive for drug targeting^57^, and multiple compounds have been synthesized towards its inhibition in the last decade, with patents issued to various pharmaceuticals including Achaogen, Astrazeneca, Merck, Pfizer and Novartis^59,90,91^. While these candidates are yet to pass human clinical trials, novel LpxC inhibitor compounds are being developed with validated antibacterial activities against *E. coli* and *P. aeruginosa*^92^, confirming that LpxC is currently still considered an attractive target to tackle Gram-negative bacteria.

Another group of high-ranking proteins identified through our prioritization pipeline participate in fatty acid synthesis (FAS) processes, and include FabA, FabB, FabD, FabI, and FabH. These proteins have an essential role during the synthesis of bacterial phospholipid membranes, LPS, and lipoproteins, and represent attractive targets due to the structural differences between the human and bacterial proteins and the essentiality of FAS^93,94^. Inhibitors of some of these proteins have been previously reported, such as platencin, a dual inhibitor of FabH (an initiation condensing enzyme) and FabF (an enoyl-ACP reductase)^95^. Efforts of developing antimicrobials targeting FAS have been geared mostly towards FabI, with two commercially available inhibitors of this enzyme, triclosan and isoniazid, the latter a first line antituberculosis drug^96,97^. The finding of FAS proteins as highly-ranked in our investigation also contributes towards validating our methodological strategy.

While previously reported drug targets were identified during our analyses, we also determined a set of proteins as both high- and intermediate-value targets displaying interesting characteristics that could be further explored for drug development, representing novel candidate targets for the control of *Kp* (and related bacteria). These include CDP-diacylglycerol–serine O-phosphatidyltransferase (*pssA*), involved in phosphatidylserine synthesis^98^, thus also participating in important transformations leading to phospholipid synthesis. Previous studies with a *pssA* mutant of *B. abortus* correlated the effect of increased sensitivity to PB in this Gram-negative pathogen^72^, which triggers a prospect of a polymyxin combination therapy with PssA inhibitors. Also, IspE that is involved in the biosynthesis of isoprenoids via the MEP pathway (absent in the human host), and for which inhibitory activities against the enzyme from *Klebsiella* and related bacteria remains to be investigated. Furthermore, protein targets FolE and FolB that participate in the folate biosynthesis pathway have not been extensively explored as drug targets in Gram-negative bacteria. A peculiar candidate is GshA that has a detoxifying role, maintaining an intracellular reducing environment. Drugs which disrupt such detoxification systems could be combined with bactericidal compounds for a more effective bacterial killing. Lastly, it is worth to mention that kinases also represent attractive candidates targets for further investigations, in line with recent reports showing that inhibitors of human kinases could be repurposed for use against bacterial enzymes. In summary, a series of biologically interesting targets that take part in distinct molecular processes and that cope with druggability features were identified in the present work.

With regards to the cellular compartment where these candidates are found, most of the top-ranked proteins locate either in the cytoplasm or in the cell membrane, and are a priori unavailable for external binding. This apparent inappropriateness of cytoplasmic proteins to serve as target should not hamper their further exploration, as it is well known that many antibiotics are capable of efficiently crossing bacterial membranes (either by diffusion or through porin channels)^99^, coupled with recent developments of delivery strategies including the use of siderophores, cyclodextrins, metal nanoparticles, antimicrobial/cell-penetrating peptides and fusogenic liposomes^100^. Thus, that a candidate target locates cytoplasmically should not detain future design of antibacterial drugs directed towards their inhibition.

Further studies are warranted to follow-up experimentally on our elicited targets, and we invite the scientific community dedicated to this subject to help pursue these goals, thus strengthening the ongoing fight against pathogenic bacteria.

## Electronic supplementary material

Supplementary Table S1, Supplementary Table S2, Supplementary Figure S1, Supplementary Figure S2

Supplementary Table S3
