# Supplementary material for: An integrative, multi-omics approach towards the prioritization of Klebsiella pneumoniae drug targets
Source: Sci Rep. 2018 Jul 17;8:10755. doi: 10.1038/s41598-018-28916-7 (PMC6050338; doi:10.1038/s41598-018-28916-7)

Supplementary Information file

An integrative, multi-omics approach towards the prioritization of *Klebsiella pneumoniae* drug targets

Pablo Ivan Pereira Ramos[1,2#], Darío Fernández Do Porto[3,4#], Esteban Lanzarotti[5], Ezequiel J. Sosa[3], Germán Burguener[3], Agustín M. Pardo[5], Cecilia C. Klein[6,7,☯], Marie-France Sagot[6,7], Ana Tereza R. de Vasconcelos[2], Ana Cristina Gales[8], Marcelo Marti[3,4,5], Adrián G. Turjanski[3,4,5*] & Marisa F. Nicolás[2*]

Affiliations:

[1] Instituto Gonçalo Moniz, Fundação Oswaldo Cruz (FIOCRUZ), Salvador, Bahia, Brazil;

[2] Laboratório Nacional de Computação Científica, Petrópolis, Rio de Janeiro, Brazil;

[3] Plataforma de Bioinformática Argentina (BIA), Instituto de Cálculo, Facultad de Ciencias Exactas y Naturales, Universidad de Buenos Aires, Buenos Aires, Argentina;

[4] Departamento de Química Biológica, Facultad de Ciencias Exactas y Naturales, Universidad de Buenos Aires, Ciudad Universitaria, Pabellón 2, C1428EHA Ciudad de Buenos Aires, Argentina

[5] Instituto de Química Biológica de la Facultad de Ciencias Exactas y Naturales (IQUIBICEN) CONICET, Ciudad Universitaria, Pabellón 2, C1428EHA Ciudad de Buenos Aires, Argentina;

[6] Inria Grenoble Rhône-Alpes, Grenoble, France;

[7] Université Claude Bernard Lyon 1, Lyon, France;

[☯] Current address: Centre for Genomic Regulation (CRG), Barcelona, Spain;

[8] Laboratório Alerta. Division of Infectious Diseases, Department of Internal Medicine. Escola Paulista de Medicina/Universidade Federal de São Paulo, São Paulo, Brazil.

[#] Equal contributors.

[*] Corresponding authors: MFN (marisa@lncc.br), AGT (adrian@qb.fcen.uba.ar)

Supplementary Table S1

Title: Complete list of 226 genomes from the Human Microbiome Project used during our analyses. Corresponding GenBank/NCBI accession numbers are shown.

**Organism**	**NCBI Assembly Accession Number**
Erysipelatoclostridium ramosum DSM 1402	GCF_000154485.1
Lactobacillus ruminis ATCC 25644	GCF_000225845.1
Clostridium] methylpentosum DSM 5476	GCF_000158655.1
Staphylococcus sp. HGB0015	GCF_000411275.1
Eggerthella lenta 1_1_60AFAA	GCF_000763035.1
Coprococcus eutactus ATCC 27759	GCF_000154425.1
Streptomyces sp. HGB0020	GCF_000411315.1
Parabacteroides goldsteinii DSM 19448 = WAL 12034	GCF_000969835.1
Corynebacterium sp. HFH0082	GCF_000411235.1
Eubacterium sp. 3_1_31	GCF_000273585.1
Clostridium perfringens WAL-14572	GCF_000243175.1
Proteus penneri ATCC 35198	GCF_000155835.1
Prevotella salivae DSM 15606	GCF_000185845.1
Blautia obeum ATCC 29174	GCF_000153905.1
Ruminococcus sp. 5_1_39BFAA	GCF_000159975.1

Erysipelotrichaceae bacterium 2_2_44A	GCF_000225685.1
Ruminococcus gnavus ATCC 29149	GCF_000169475.1
Anaerostipes caccae DSM 14662	GCF_000154305.1
Parvimonas micra ATCC 33270	GCF_000154405.1
Alistipes putredinis DSM 17216	GCF_000154465.1
Bacteroides cellulosilyticus DSM 14838	GCF_000158035.1
Edwardsiella tarda ATCC 23685	GCF_000163955.1
Helicobacter pullorum MIT 98-5489	GCF_000155495.1
Bacteroides] pectinophilus ATCC 43243	GCF_000155855.1
Bacteroides caccae ATCC 43185	GCF_000169015.1
Helicobacter bilis ATCC 43879	GCF_000158435.1
Faecalibacterium prausnitzii M21/2	GCF_000154385.1
Lactobacillus brevis subsp. gravesensis ATCC 27305	GCF_000159175.1
Lactobacillus rhamnosus LMS2-1	GCF_000160175.1
Bacteroides fluxus YIT 12057	GCF_000195635.1
Bifidobacterium dentium ATCC 27678	GCF_000172135.1
Enterobacteriaceae bacterium 9_2_54FAA	GCF_000185685.2
Dialister succinatiphilus YIT 11850	GCF_000242435.1
Veillonella sp. 6_1_27	GCF_000163735.1

Bacillus smithii 7_3_47FAA	GCF_000238675.1
Campylobacter coli JV20	GCF_000146835.1
Bilophila wadsworthia 3_1_6	GCF_000185705.2
Parabacteroides johnsonii DSM 18315	GCF_000156495.1
Helicobacter canadensis MIT 98-5491	GCF_000162575.1
Desulfovibrio piger ATCC 29098	GCF_000156375.1
Dysgonomonas gadei ATCC BAA-286	GCF_000213555.1
Anaerobaculum hydrogeniformans ATCC BAA-1850	GCF_000160455.2
Anaerostipes hadrus DSM 3319	GCF_000332875.2
Escherichia coli MS 85-1	GCF_000179075.1
Leuconostoc mesenteroides subsp. cremoris ATCC 19254	GCF_000160595.1
Phascolarctobacterium succinatutens YIT 12067	GCF_000188175.1
Clostridium] bolteae ATCC BAA-613	GCF_000154365.1
Turicibacter sp. HGF1	GCF_000191865.1
Collinsella intestinalis DSM 13280	GCF_000156175.1
Bacteroides xylanisolvens SD CC 1b	GCF_000178215.1
Bacteroides intestinalis DSM 17393	GCF_000172175.1
Catenibacterium mitsuokai DSM 15897	GCF_000173795.1
Dorea sp. D27	GCF_001185345.1

Parabacteroides sp. HGS0025	GCF_000969845.1
Bifidobacterium breve DSM 20213 = JCM 1192	GCF_001311295.1
Streptococcus anginosus 1_2_62CV	GCF_000186545.1
Clostridium] spiroforme DSM 1552	GCF_000154805.1
Helicobacter cinaedi CCUG 18818 = ATCC BAA-847	GCF_000349975.1
Subdoligranulum sp. 4_3_54A2FAA	GCF_000238635.1
Citrobacter youngae ATCC 29220	GCF_000155975.1
Campylobacter sp. 10_1_50	GCF_000238755.1
Bifidobacterium sp. 12_1_47BFAA	GCF_000185665.1
Sutterella parvirubra YIT 11816	GCF_000250875.1
Bacteroides stercoris ATCC 43183	GCF_000154525.1
Ruminococcus callidus ATCC 27760	GCF_000468015.1
Desulfovibrio sp. 6_1_46AFAA	GCF_000224635.1
Eubacterium] siraeum DSM 15702	GCF_000382085.1
Holdemanella biformis DSM 3989	GCF_000156655.1
Providencia alcalifaciens F90-2004	GCF_000517805.1
Ralstonia sp. 5_2_56FAA	GCF_000227255.2
Bacteroides fragilis 3_1_12	GCF_000157015.1
Dermabacter sp. HFH0086	GCF_000413375.1

Eubacterium] hallii DSM 3353	GCF_000173975.1
Enterococcus saccharolyticus 30_1	GCF_000234175.1
Paenibacillus sp. HGF5	GCF_000204455.1
Blautia hansenii DSM 20583	GCF_002222595.1
Helicobacter macacae MIT 99-5501	GCF_000507845.1
Campylobacter upsaliensis JV21	GCF_000185345.1
Synergistes sp. 3_1_syn1	GCF_000238615.1
Coprococcus comes ATCC 27758	GCF_000155875.1
Bacteroides clarus YIT 12056	GCF_900129655.1
Clostridium] symbiosum ATCC 14940	GCF_000466485.1
Collinsella aerofaciens ATCC 25986	GCF_000169035.1
Lactobacillus amylolyticus DSM 11664	GCF_001435665.1
Dorea formicigenerans 4_6_53AFAA	GCF_000225745.1
Bacteroides plebeius DSM 17135	GCF_000187895.1
Eubacterium ventriosum ATCC 27560	GCF_000153885.1
Fusobacterium nucleatum subsp. animalis ATCC 51191	GCF_000220825.1
Providencia rettgeri DSM 1131	GCF_000158055.1
Prevotella oralis HGA0225	GCF_000413355.1
Lactobacillus ultunensis DSM 16047	GCF_001436305.1

Prevotella copri DSM 18205	GCF_000157935.1
Fusobacterium necrophorum subsp. funduliforme 1_1_36S	GCF_000242215.1
Clostridium] hylemonae DSM 15053	GCF_000156515.1
Bacteroides coprophilus DSM 18228 = JCM 13818	GCF_001315785.1
Methanobrevibacter smithii DSM 2375	GCF_000151245.1
Clostridium] leptum DSM 753	GCF_000154345.1
Anaerofustis stercorihominis DSM 17244	GCF_000154825.1
Dysgonomonas mossii DSM 22836	GCF_000376405.1
Providencia rustigianii DSM 4541	GCF_000156395.1
Lactobacillus hilgardii DSM 20176 = ATCC 8290	GCF_001434655.1
Tannerella sp. 6_1_58FAA_CT1	GCF_000238695.1
Helicobacter winghamensis ATCC BAA-430	GCF_000158455.1
Collinsella tanakaei YIT 12063	GCF_000225705.1
Subdoligranulum variabile DSM 15176	GCF_000157955.1
Odoribacter laneus YIT 12061	GCF_000243215.1
Collinsella stercoris DSM 13279	GCF_000156215.1
Streptococcus equinus ATCC 9812	GCF_000187265.1
Clostridium citroniae WAL-17108	GCF_000233455.1
Clostridium clostridioforme 2_1_49FAA	GCF_000234155.1

Pseudomonas sp. 2_1_26	GCF_000233495.1
Proteus mirabilis WGLW6	GCF_000297815.1
Flavonifractor plautii ATCC 29863	GCF_000239295.1
Bifidobacterium adolescentis L2-32	GCF_000154085.1
Propionibacterium sp. HGH0353	GCF_000413335.1
Clostridium] scindens ATCC 35704	GCF_000154505.1
Enterococcus faecalis 918	GCF_000690925.1
Paraprevotella clara YIT 11840	GCF_000233955.1
Lactobacillus plantarum subsp. plantarum ATCC 14917 = JCM 1149 = CGMCC 1.2437	GCF_001434175.1
Paraprevotella xylaniphila YIT 11841	GCF_000205165.1
Listeria grayi DSM 20601	GCF_000148995.1
Anaerotruncus colihominis DSM 17241	GCF_000154565.1
Eggerthella sp. HGA1	GCF_000191845.1
Bacillus sp. 7_6_55CFAA_CT2	GCF_000238655.1
Eubacterium] cylindroides ATCC 27803	GCF_000469305.1
Coprococcus sp. HPP0074	GCF_000411335.1
Slackia piriformis YIT 12062	GCF_000296445.1
Bifidobacterium gallicum DSM 20093 = LMG 11596	GCF_000771165.1
Bifidobacterium pseudocatenulatum DSM 20438 = JCM 1200 = LMG 10505	GCF_001025215.1

Lactobacillus helveticus DSM 20075 = CGMCC 1.1877	GCF_001434945.1
Streptococcus infantarius subsp. infantarius ATCC BAA-102	GCF_000154985.1
Collinsella sp. 4_8_47FAA	GCF_000763055.1
Alistipes indistinctus YIT 12060	GCF_000231275.1
Bacteroides uniformis ATCC 8492	GCF_000154205.1
Clostridium orbiscindens 1_3_50AFAA	GCF_000760655.1
Intestinibacter bartlettii DSM 16795	GCF_900167285.1
Butyricicoccus pullicaecorum 1.2	GCF_000398925.1
Barnesiella intestinihominis YIT 11860	GCF_000296465.1
Oscillibacter sp. KLE 1745	GCF_000469445.2
Acinetobacter junii SH205	GCF_000162075.1
Listeria innocua ATCC 33091	GCF_000241405.1
Clostridium asparagiforme] DSM 15981	GCF_000158075.1
Bilophila sp. 4_1_30	GCF_000224655.1
Corynebacterium ammoniagenes DSM 20306	GCF_001941425.1
Enterobacter cancerogenus ATCC 35316	GCF_000155995.1
Lachnospiraceae bacterium 3_1_57FAA_CT1	GCF_000218405.2
Bacteroides coprocola DSM 17136	GCF_000154845.1
Eubacterium ramulus ATCC 29099	GCF_000469345.1

Coprobacillus sp. 3_3_56FAA	GCF_000239735.1
Bacteroides sp. 2_2_4	GCF_000157055.1
Pediococcus acidilactici 7_4	GCF_000163095.1
Dorea longicatena DSM 13814	GCF_000154065.1
Aneurinibacillus aneurinilyticus ATCC 12856	GCF_000466385.1
Cetobacterium somerae ATCC BAA-474	GCF_000479045.1
Butyrivibrio crossotus DSM 2876	GCF_000156015.1
Oxalobacter formigenes HOxBLS	GCF_000158475.1
Lactobacillus buchneri ATCC 11577	GCF_000159195.1
Pseudoflavonifractor capillosus ATCC 29799	GCF_000169255.2
Bacteroides eggerthii 1_2_48FAA	GCF_000273465.1
Blautia hydrogenotrophica DSM 10507	GCF_000157975.1
Fusobacterium varium ATCC 27725	GCF_000159915.2
Lactobacillus fermentum ATCC 14931	GCF_000159215.1
Roseburia intestinalis L1-82	GCF_000156535.1
Streptococcus sp. 2_1_36FAA	GCF_000161955.1
Clostridium celatum DSM 1785	GCF_000320405.1
Ruminococcaceae bacterium D16	GCF_000177015.2
Bifidobacterium bifidum ATCC 29521 = JCM 1255 = DSM 20456	GCF_001025135.1

Peptoclostridium difficile NAP08	GCF_000164175.1
Mitsuokella multacida DSM 20544	GCF_000155955.1
Ruminococcus lactaris ATCC 29176	GCF_000155205.1
Ruminococcus torques ATCC 27756	GCF_000153925.1
Clostridium sporogenes ATCC 15579	GCF_000155085.1
Succinatimonas hippei YIT 12066	GCF_000188195.1
Holdemania filiformis DSM 12042	GCF_000157995.1
Bacteroides oleiciplenus YIT 12058	GCF_000315485.1
Lactobacillus delbrueckii subsp. lactis DSM 20072	GCA_002017855.1
Yokenella regensburgei ATCC 43003	GCF_000239335.1
Bifidobacterium angulatum DSM 20098 = JCM 7096	GCF_001025155.1
Tyzzerella nexilis DSM 1787	GCF_000156035.2
Porphyromonas sp. 31_2	GCA_000712235.1
Roseburia inulinivorans DSM 16841	GCF_000174195.1
Bifidobacterium catenulatum DSM 16992 = JCM 1194 = LMG 11043	GCF_001025195.1
Escherichia sp. 3_2_53FAA	GCF_000157115.2
Parasutterella excrementihominis YIT 11859	GCF_000205025.1
Arcobacter butzleri JV22	GCF_000185325.1
Helicobacter pylori GAM115Ai	GCF_000345045.1

Bacteroides salyersiae WAL 10018 = DSM 18765 = JCM 12988	GCF_000381365.1
Clostridiales bacterium 1_7_47FAA	GCF_000155435.1
Clostridium sp. KLE 1755	GCF_000466465.2
Bacteroides dorei DSM 17855	GCF_000156075.1
Cedecea davisae DSM 4568	GCF_000412335.1
Clostridium] hiranonis DSM 13275	GCF_000156055.1
Hafnia alvei ATCC 51873	GCF_000239255.1
Weissella paramesenteroides ATCC 33313	GCF_000160575.1
Prevotella stercorea DSM 18206	GCF_000235885.1
Eubacterium] dolichum DSM 3991	GCF_000154285.1
Acidaminococcus sp. D21	GCF_000174215.1
Coprobacillus sp. D7	GCF_000158555.1
Anaerococcus hydrogenalis DSM 7454	GCF_000173355.1
Lactobacillus paracasei subsp. paracasei ATCC 25302	GCF_000159495.1
Clostridium] hathewayi DSM 13479	GCF_000160095.1
Citrobacter sp. 30_2	GCA_002215605.1
Megamonas funiformis YIT 11815	GCF_000245775.1
Paenisporosarcina sp. HGH0030	GCF_000411295.1
Blautia sp. KLE 1732	GCF_000466565.1

Bacteroides vulgatus PC510	GCF_000178195.1
Fusobacterium mortiferum ATCC 9817	GCF_000158195.2
Marvinbryantia formatexigens DSM 14469	GCF_000173815.1
Fusobacterium ulcerans ATCC 49185	GCF_000158315.2
Neisseria macacae ATCC 33926	GCF_000220865.1
Lactobacillus antri DSM 16041	GCF_001435475.1
Lactobacillus sp. 7_1_47FAA	GCF_000227195.1
Helicobacter canis NCTC 12740	GCF_000507865.1
Lactobacillus acidophilus ATCC 4796	GCF_000159715.1
Parabacteroides merdae ATCC 43184	GCF_000154105.1
Desulfitobacterium hafniense DP7	GCF_000238035.1
Acinetobacter radioresistens SH164	GCF_000162115.1
Burkholderiales bacterium 1_1_47	GCF_000144975.1
Citrobacter freundii 4_7_47CFAA	GCF_000238735.1
Bacteroides ovatus 3_8_47FAA	GCF_000218325.1
Bifidobacterium longum subsp. longum 2-2B	GCF_000261205.1
Fusobacterium gonidiaformans ATCC 25563	GCF_000158835.2
Bacteroides finegoldii DSM 17565	GCF_000156195.1
Sutterella wadsworthensis 3_1_45B	GCF_000186505.1

Actinomyces sp. HPA0247	GCF_000411415.1
Providencia stuartii ATCC 25827	GCF_000154865.1

Supplementary Table S2

Title: Genomes of pathogenic *Klebsiella pneumoniae* and corresponding NCBI/GenBank accession numbers used in conservation analysis.

**Organism**	**Strain**	**NCBI Assembly Accession Number**
Klebsiella pneumoniae subsp. pneumoniae	HS11286	GCA_000240185.2
Klebsiella pneumoniae	ATCC BAA-2146	GCA_000364385.2
Klebsiella pneumoniae	JM45	GCA_000445405.1
Klebsiella pneumoniae subsp. pneumoniae	PittNDM01	GCA_000733255.1
Klebsiella pneumoniae	PMK1	GCA_000764615.1
Klebsiella pneumoniae subsp. pneumoniae	KPNIH29	GCA_000784945.1
Klebsiella pneumoniae	XH209	GCA_000775955.1
Klebsiella pneumoniae subsp. pneumoniae	NTUH-K2044	GCA_000009885.1
Klebsiella pneumoniae subsp. pneumoniae	1084	GCA_000294365.1
Klebsiella pneumoniae subsp. pneumoniae	KPNIH10	GCA_000281435.2
Klebsiella pneumoniae subsp. pneumoniae	KPNIH1	GCA_000281535.2

Klebsiella pneumoniae	500_1420	GCA_000406765.2
Klebsiella pneumoniae	UHKPC33	GCA_000417085.2
Klebsiella pneumoniae	DMC1097	GCA_000417225.2
Klebsiella pneumoniae	UHKPC07	GCA_000417265.2
Klebsiella pneumoniae	30684/NJST258_2	GCA_000597905.1
Klebsiella pneumoniae	30660/NJST258_1	GCA_000598005.1
Klebsiella pneumoniae subsp. pneumoniae	KPNIH24	GCA_000714675.1
Klebsiella pneumoniae subsp. pneumoniae	KPR0928	GCA_000717515.1
Klebsiella pneumoniae subsp. pneumoniae	KPNIH33	GCA_000775375.1
Klebsiella pneumoniae subsp. pneumoniae	KPNIH32	GCA_000775395.1
Klebsiella pneumoniae subsp. pneumoniae	KPNIH30	GCA_000784985.1
Klebsiella pneumoniae	CAV1596	GCA_001022235.1
Klebsiella pneumoniae subsp. pneumoniae	KPNIH27	GCA_000695935.1
Klebsiella pneumoniae subsp. pneumoniae	ATCC 700721; MGH 78578	GCA_000016305.1
Klebsiella pneumoniae subsp. pneumoniae	KPNIH31	GCA_000785005.1

Klebsiella pneumoniae	blaNDM-1	GCA_000739495.1
Klebsiella pneumoniae subsp. pneumoniae	Kp13	GCA_000512165.1
Klebsiella pneumoniae subsp. pneumoniae	ATCC 43816 KPPR1	GCA_000742755.1
Klebsiella pneumoniae subsp. pneumoniae	234-12	GCA_000981845.1
Klebsiella pneumoniae	Kp52.145	GCA_000968155.1
Klebsiella pneumoniae	CG43	GCA_000474015.1
Klebsiella pneumoniae	CAV1344	GCA_001022175.1
Klebsiella pneumoniae	CAV1392	GCA_001022035.1
Klebsiella pneumoniae	32192	GCA_000807395.3
Klebsiella pneumoniae	HK787	GCA_000813205.1
Klebsiella pneumoniae	34618	GCA_000814305.1
Klebsiella pneumoniae subsp. pneumoniae	1158	GCA_000814805.1

Supplementary Figure S1

Title: An overview of the prioritization process.

Description: An overview of the total proteome count for *Klebsiella pneumoniae* Kp13 and the subsequent shortlisting steps, which included the obtaining of structural models and identification of druggable features therein. The 2,769 proteins in the golden set were those ranked according to Equations 1, 2 and 3 (main manuscript text).

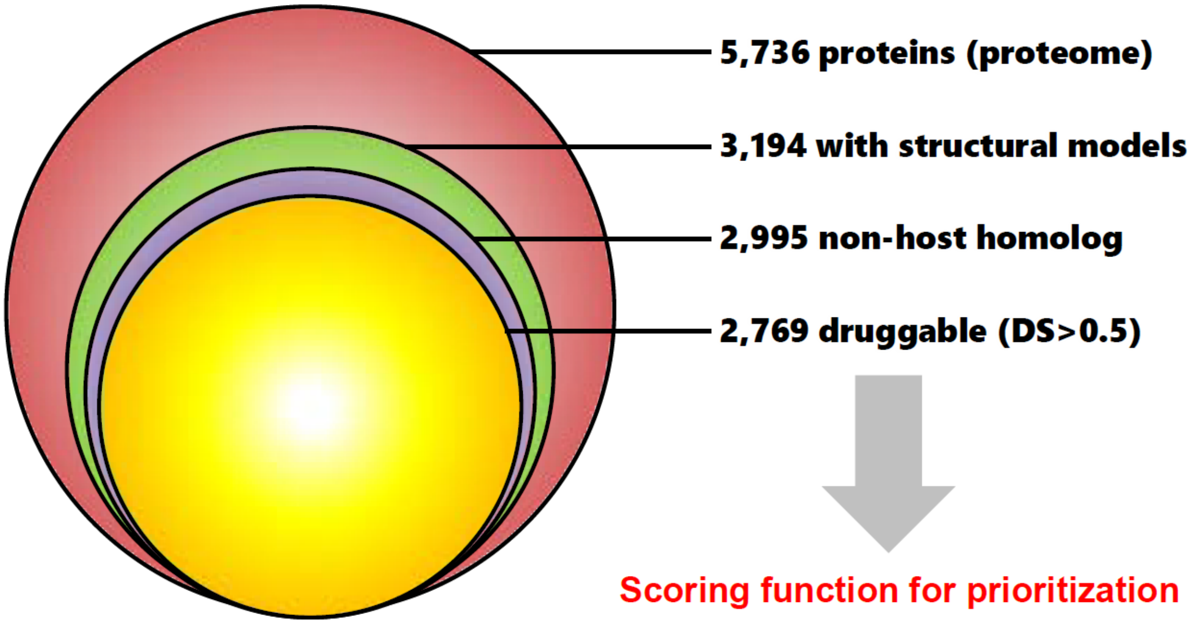

Supplementary Figure S2

Title: Protein conservation of *K. pneumoniae* Kp13 (reference) compared to other pathogenic strains (lines).

Description: Each red-colored cell indicate the presence of an ortholog in *K. pneumoniae* Kp13 in the compared strain (see Methods for sequence comparison criteria). No ordering was performed along the X-axis, which follows Kp13 increasing locus ordering. Hierarchical clustering was performed along the Y-axis indicating relationship between the strains based on ortholog presence/absence. The blue bar above the image indicates chromosomally-encoded sequences, while the yellow bar indicates plasmidial-encoded sequences, indicating that conservation of chromosomal sequences is much larger than those encoded by genes in plasmidial genes.

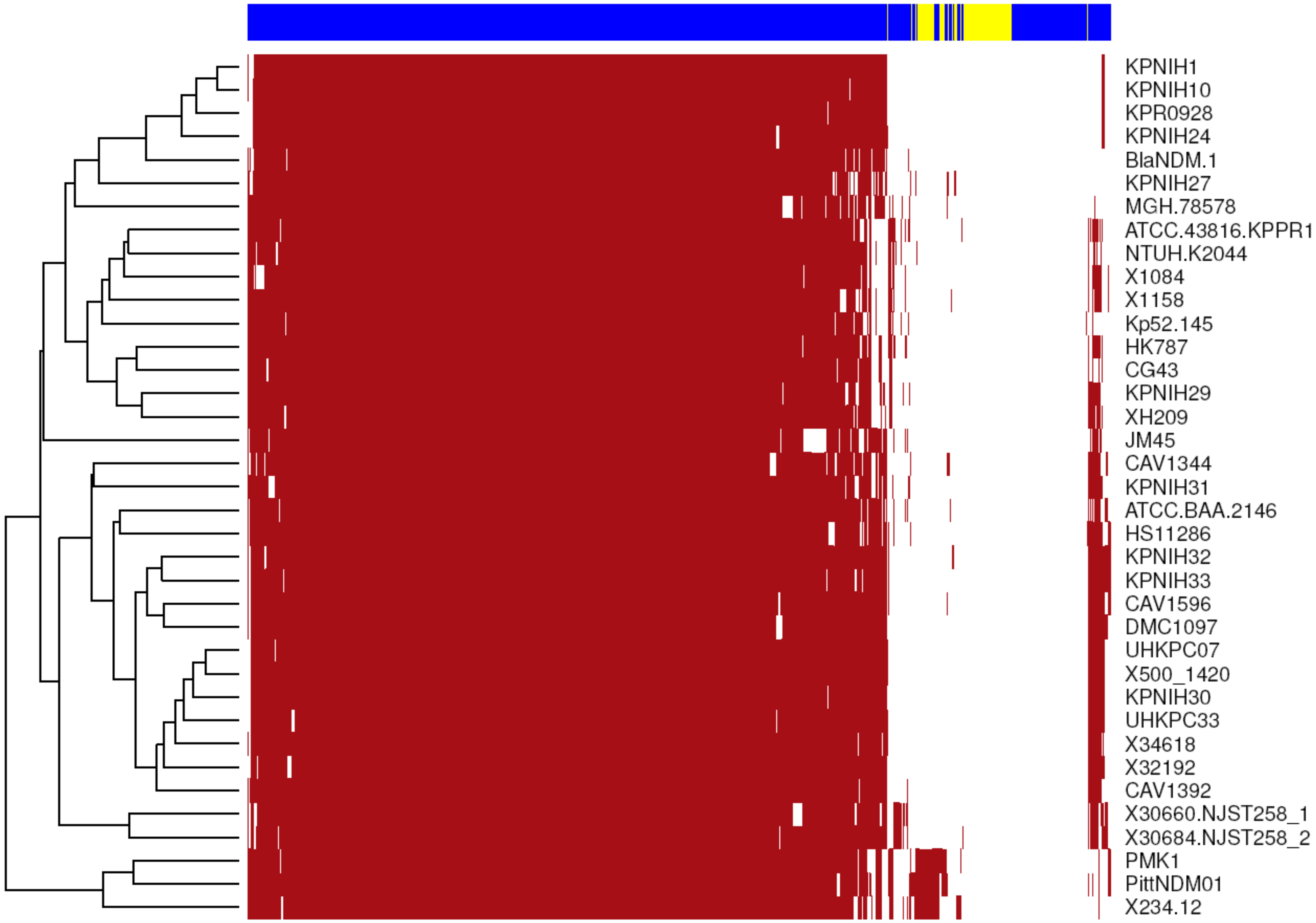

Supplement: Supplementary file 1 — Supplementary Table S1, Supplementary Table S2, Supplementary Figure S1, Supplementary Figure S2 [file 41598_2018_28916_MOESM1_ESM.pdf]
